# Analysis of In Vivo Existence Forms of Nardosinone in Mice by UHPLC-Q-TOF-MS Technique

**DOI:** 10.3390/molecules27217267

**Published:** 2022-10-26

**Authors:** Jing Zhang, Yang Lv, Jing Zhang, Yu-Sha Bai, Meng-Yuan Li, Shun-Qi Wang, Li-Li Wang, Guang-Xue Liu, Feng Xu, Ming-Ying Shang, Shao-Qing Cai

**Affiliations:** 1 State Key Laboratory of Natural and Biomimetic Drugs, School of Pharmaceutical Sciences, Peking University, No. 38 Xueyuan Road, Beijing 100191, China; 2 School of Pharmacy, Henan University of Chinese Medicine, No. 156 Jinshui East Road, Zhengzhou 450046, China

**Keywords:** nardosinone, existence form, in vivo metabolism, UHPLC-Q-TOF-MS, sesquiterpene peroxide

## Abstract

Nardosinone, a sesquiterpene peroxide, is one of the main active constituents of the ethnomedicine Nardostachyos Radix et Rhizoma, and it has many bioactivities, such as antiarrhythmia and cardioprotection. To elucidate its in vivo existence forms, its metabolism is first studied using mice. All urine and feces are collected during the six days of oral dosing of nardosinone, and blood is collected at one hour after the last dose. Besides, to validate some metabolites, a fast experiment is performed, in which nardosinone was orally administered and the subsequent one-hour urine is collected and immediately analyzed by UHPLC-Q-TOF-MS. In total, 76 new metabolites are identified in this study, including 39, 51, and 12 metabolites in urine, plasma, and feces, respectively. Nardosinone can be converted into nardosinone acid or its isomers. The metabolic reactions of nardosinone included hydroxylation, hydrogenation, dehydration, glucuronidation, sulfation, demethylation, and carboxylation. There are 56 and 20 metabolites with the structural skeleton of nardosinone and nardosinone acid, respectively. In total, 77 in vivo existence forms of nardosinone are found in mice. Nardosinone is mainly excreted in urine and is not detected in the feces. These findings will lay the foundation for further research of the in vivo effective forms of nardosinone and Nardostachyos Radix et Rhizoma.

## 1. Introduction

Nardosinone is a sesquiterpene peroxide and exists in a variety of medicinal plants, such as *Nardostachys jatamansi* DC. [1], *Millettia speciosa* [2], *Sargassum fusiforme* [3], *Taraxacum kok-saghyz* Rodin [4], *Chrysanthemum morifolium* [5]. In addition, nardosinone was also detected in Mahuang Fuzi Xixin decoction [6]. Nardosinone is mainly derived from *Nardostachys jatamansi* DC. Nardostachyos Radix et Rhizoma (NRR), the root and rhizome of *Nardostachys jatamansi* DC., has the traditional pharmacological action of regulating qi, relieving pain, and relieving stagnation-syndrome, and is often used as the raw material for spices, food and cosmetics [7], and contains multiple chemical constituents, such as terpenes, flavonoids, coumarins, lignans, and volatile oils [8], of which sesquiterpenoids (e.g., nardosinone) are the main active components [9]. Pharmacological studies have shown that NRR has pharmacological effects of improving headaches, palpitations, and syncope [9], and it also has antioxidant, neuroprotective, antiepileptic, cardioprotective, anti-neuroinflammation, and other pharmacological activities [10,11,12]. Furthermore, NRR is mainly used in the form of compound prescription in clinical, e.g., Wenxin Granules can be used to treat premature ventricular contractions in patients with chronic heart failure [1].

Modern pharmacological studies have shown that nardosinone could exert anti-inflammatory, neuroprotective, cardioprotective, and more pharmacological actions [1]. For example, it could play a role in antiarrhythmia by hindering the calcium overload in the cAMP/PKA signaling pathway [13]. Nardosinone can regulate the proliferation and differentiation of neural stem cells, which implies that it might have therapeutic effects on brain injury and neurodegenerative diseases [14]. Nardosinone could also exert a cardioprotective effect, such as inhibiting the hypertrophy of H9c2 cells induced by Ang II [15]. Moreover, nardosinone, as the most effective anti-Parkinson’s disease compound in NRR, has been verified that it could alleviate Parkinson’s disease symptoms in mice [16]. It was manifested that nardosinone had good anti-trypanosomal activity in vitro [17]. However, nardosinone is easily degraded and generated other constitutes in the conditions of light and high temperature, while it was stable in alkaline conditions and low temperature. Therefore, it should be noted that the temperature should be controlled below 40 °C, the pH of the solution should be controlled between 7 and 12, and light should be avoided in the experimental process of extraction, separation, and administration of nardosinone [18].

Natural peroxides have multiple biological activities and are one of the important natural product types. For example, a large number of chemical components, such as polyketones with six-membered or five-membered peroxide rings, have been found in sponges [19,20,21], and the great majority of components have pharmacological effects such as anticancer, antibacterial, and antimalarial [21]; two diterpenoids with seven-membered peroxy rings were found in rhizomes of *Hedychium coronarium* [22]; a peroxydimer monoterpene was isolated from *Amomum cardamomum*, which has powerful antimalarial activity [23]. Furthermore, there were also a large number of studies on sesquiterpene peroxides, such as artemisinin compounds (artemisinin, dihydroartemisinin, and artemether). Studies have demonstrated that the peroxy-bond was the key pharmacophore of the antimalarial effect of artemisinin [24], and the peroxy-bond could be broken into monooxygen bridges based on the metabolism studies of artemisinin and dihydroartemisinin in vivo and in vitro [25]. Yingzhaosu A, as a sesquiterpene peroxide, also has significant antimalarial activity [26,27], but there are no reports on its metabolism research. The sesquiterpene peroxide (1R, 4R, 6S, 7S, 9S)-4α-hydroxy-1,9-peroxybisabola-2,10-diene was isolated from the rhizomes of *Alpinia japonica* [28]; the sesquiterpene peroxide 1α,8α-epidioxy-4α-hydroxy-5αH-guai-7(11),9-dien-12,8-olide, which has good anti-influenza virus activity was isolated from *Curcuma wenyujin* [29]. The activity of sesquiterpene peroxides has been studied in-depth, while the metabolism researches of them are less, hence their metabolic pathways and existence forms in vivo are still unclear.

Therefore, the metabolism of nardosinone in mice was carried out to (1) clarify the metabolites and metabolic pathways of nardosinone in mice; (2) elucidate whether the peroxy-bond of nardosinone is broken to form a mono-oxygen bridge in vivo and whether nardosinone can lose hydroxy-isopropyl in vivo to generate nardosinone acid or its isomers. This study will help with the following: (1) reveal the existing forms of nardosinone in vivo, and lay the foundation for further searching for the effective forms; (2) further illustrate the metabolic characteristics of sesquiterpene peroxide; (3) lay the foundation for exploring the effective forms of Nardostachyos Radix et Rhizoma.

## 2. Results

**Figure 1:**
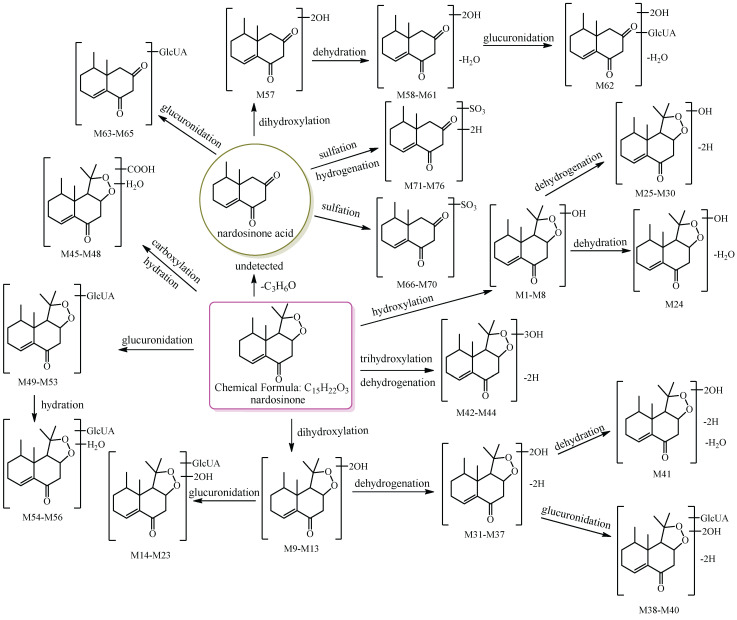
The 76 new metabolites and proposed metabolic pathway of nardosinone in mice. The pink rim and olive circle represent nardosinone and nardosinone acid, respectively.

In this study, 39, 51, and 12 metabolites were respectively identified in urine, plasma, and feces in mice (Figures S1–S3), and a total of 76 new metabolites (Table 1) were detected and preliminarily identified and the metabolic pathway of nardosinone is shown in Figure 1. Nardosinone could lose hydroxy-isopropyl to generate nardosinone acid or its isomers in the metabolism in vivo, which is a rare metabolic reaction. There were 56 and 20 metabolites with the structural skeleton of nardosinone and nardosinone acid, respectively.

**Figure 2:**
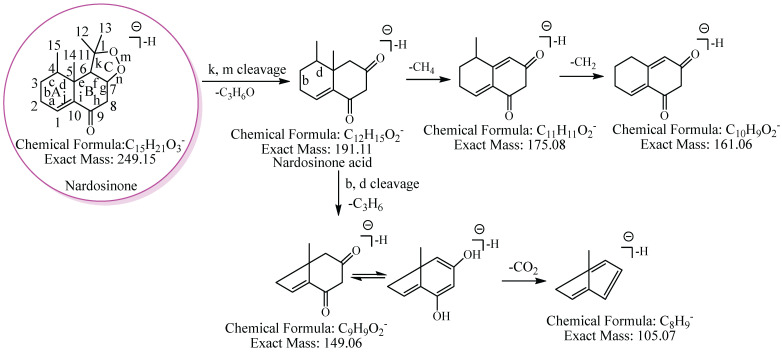
The proposed fragmentation pathway of nardosinone. The pink circle represents nardosinone, and the C-C bonds and carbon atoms in the skeleton were designated by the letters a–n and numbers 1–15; A, B and C represent the three rings, respectively.

To conveniently describe the fragment characteristics of nardosinone and its metabolites, the three rings were named A, B, and C, respectively. The C-C bonds and carbon atoms in the skeleton were designated by the letters a-n and numbers 1–15 (Figure 2).

In this study, the usual neutral losses in mass spectrometry were 58.04 Da (C_3_H_6_O), 30.01 Da (CH_2_O), 14.02 Da (CH_2_), 16.03 Da (CH_4_), 42.05 Da (C_3_H_6_), 176.03 Da (C_6_H_8_O_6_), 79.95 Da (SO_3_), 43.99 Da (CO_2_), 27.99 Da (CO), 42.01 Da (C_2_H_2_O), 122.04 Da (C_7_H_6_O_2_), and 162.05 Da (C_6_H_10_O_5_), indicating that a molecule which shows these neutral losses may contain hydroxy-isopropyl, formaldehyde or methanol, methyl, methane, propyl group, glucuronyl, sulfonyl, carboxyl or lactone, carbonyl, acetyl, benzoyl, and hexosyl (more likely glucosyl) groups, respectively. In addition, the fragment ions at *m*/*z* 175.02 (C_6_H_7_O_6_), *m*/*z* 193.04 (C_6_H_9_O_7_), *m*/*z* 96.96 (SO_4_H), *m*/*z* 81.97 Da (H_2_SO_3_), and *m*/*z* 191.11 (C_12_H_15_O_2_) indicate that the molecule may contain glucuronyl, glucuronic acid, sulfate, sulfonyl, and nardosinone acid groups, respectively.

### 2.1. Mass Spectral Fragmentation Features of Nardosinone

The proposed fragmentation pathway of nardosinone is shown in Figure 2. Nardosinone (C_15_H_22_O_3_) showed [M−H]^−^ at *m*/*z* 249.15. The fragment ion at *m*/*z* 191.11 (C_12_H_15_O_2_) was formed by a neutral loss of 58.04 Da (C_3_H_6_O) from the cleavage of the peroxy-bridge of the ion at *m*/*z* 249.15; the fragment ions at *m*/*z* 175.08 and *m*/*z* 161.06 were formed by the continuous neutral losses of 16.03 Da (CH_4_) and 14.02 Da (CH_2_) from the ion at *m*/*z* 191.11. And the ion at *m*/*z* 149.06, which could lose 43.99 Da (CO_2_) to form the fragment ion at *m*/*z* 105.07, and the fragmentation pathway of CO_2_ was reported [30], was formed by a neutral loss of 42.05 Da (C_3_H_6_) from the cleavage of b and d bonds of the A ring of the ion at *m*/*z* 191.11.

### 2.2. Identification of the Metabolites with the Skeleton of Nardosinone

#### 2.2.1. Metabolites Formed by Monohydroxylation of Nardosinone

M1−M8 showed [M−H]^−^ at *m*/*z* 265.14, and their molecular formulae were predicted as C_15_H_22_O_4_. There was an additional O atom in their molecular formulae in comparison with nardosinone. Therefore, they were identified as monohydroxylated nardosinones.

**Figure 3:**
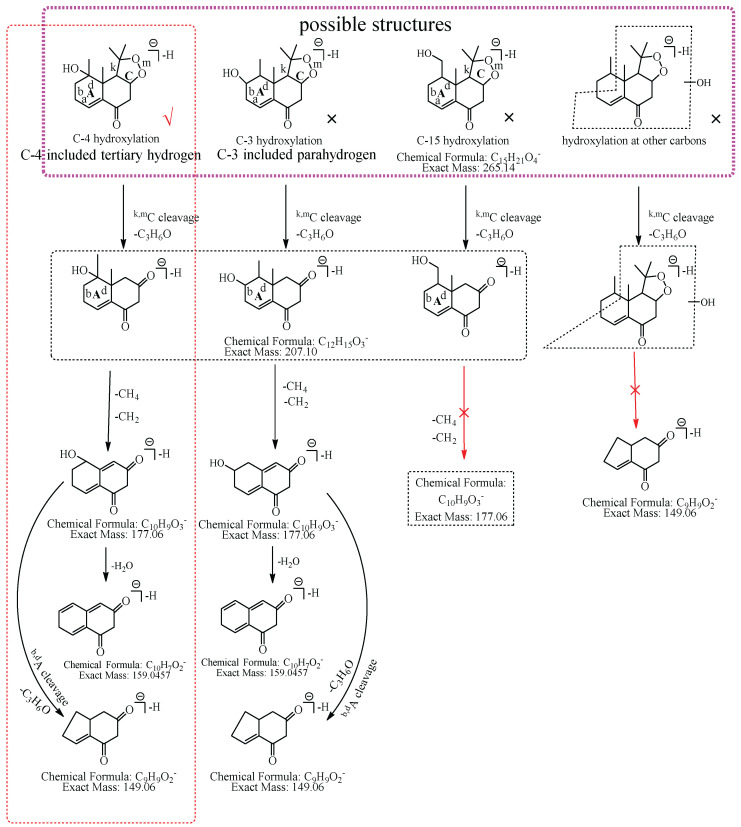
The proposed fragmentation pathways of M1 (4-hydroxyl nardosinone). “√” represents the possible structure. “×” represents the impossible structure. The purple rim represents the possible structures; A and C represent the hexatomic rings, respectively. The red rim represents the most possible fragmentation pathway.

M1: The ions at *m*/*z* 207.10 (C_12_H_15_O_3_) and *m*/*z* 149.06 (C_9_H_9_O_2_) were formed by the consecutive losses of two 58.04 Da (C_3_H_6_O) from [M−H]^−^ in the MS^2^ spectra. According to the fragmentation pathway of nardosinone, it was speculated that the cleavage of the peroxide bridge could lose C_3_H_6_O. In addition, C_3_H_6_O can be lost by the cleavage of the b and d bonds of the A ring when the hydroxylation occurs at C-3, C-4, or C-15. The ions at *m*/*z* 191.07 (C_11_H_11_O_3_) and *m*/*z* 177.06 (C_10_H_9_O_3_) were formed by the consecutive neutral losses of 16.03 Da (CH_4_) and 14.02 Da (CH_2_) from the ion at *m*/*z* 207.10, which could imply that hydroxylation did not occur at C-15. C-3 was a secondary carbon with parahydrogen, and C-4 was a tertiary carbon with tertiary hydrogen, which was more active than the parahydrogen of C-3, so it was inferred that the hydroxylation was more likely to occur at C-4. Thus, M1 was preliminarily identified as 4-hydroxyl nardosinone. The possible structure and fragmentation pathways of M1 are shown in Figure 3.

**Figure 4:**
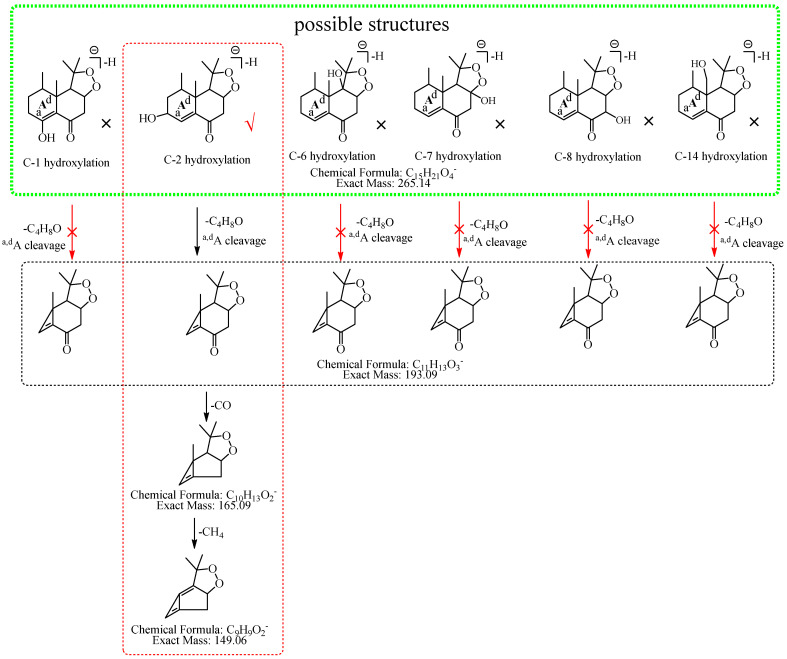
The proposed fragmentation pathways of M2 (2-hydroxyl nardosinone). “√” represents the possible structure. “×” represents the impossible structure. The green rim represents the possible structures; A represents the hexatomic ring. The red rim represents the most possible fragmentation pathway.

M2: The [M−H]^−^ of M2 directly lost 72.06 Da (C_4_H_8_O) to produce *m*/*z* 193.09, while the ion at *m*/*z* 207.10 (C_12_H_15_O_3_), which was formed by the loss of 58.04 Da (C_3_H_6_O) from [M−H]^−^ was not observed in MS^2^ spectra, demonstrating that the hydroxylation did not occur at C-3, C-4, or C-15 but it should occur at C-2. The ion at *m*/*z* 193.09 (C_11_H_13_O_3_) consecutively lost CO and 16.03 Da (CH_4_) to generate *m*/*z* 165.10 (C_10_H_13_O_2_) and *m*/*z* 149.06 (C_9_H_9_O_2_), and *m*/*z* 149.06 (C_9_H_9_O_2_) lost CO_2_ to generate *m*/*z* 105.07 (C_8_H_10_). Therefore, M2 was tentatively identified as 2-hydroxyl nardosinone. The possible structures and fragmentation pathways of M2 are shown in Figure 4.

**Figure 5:**
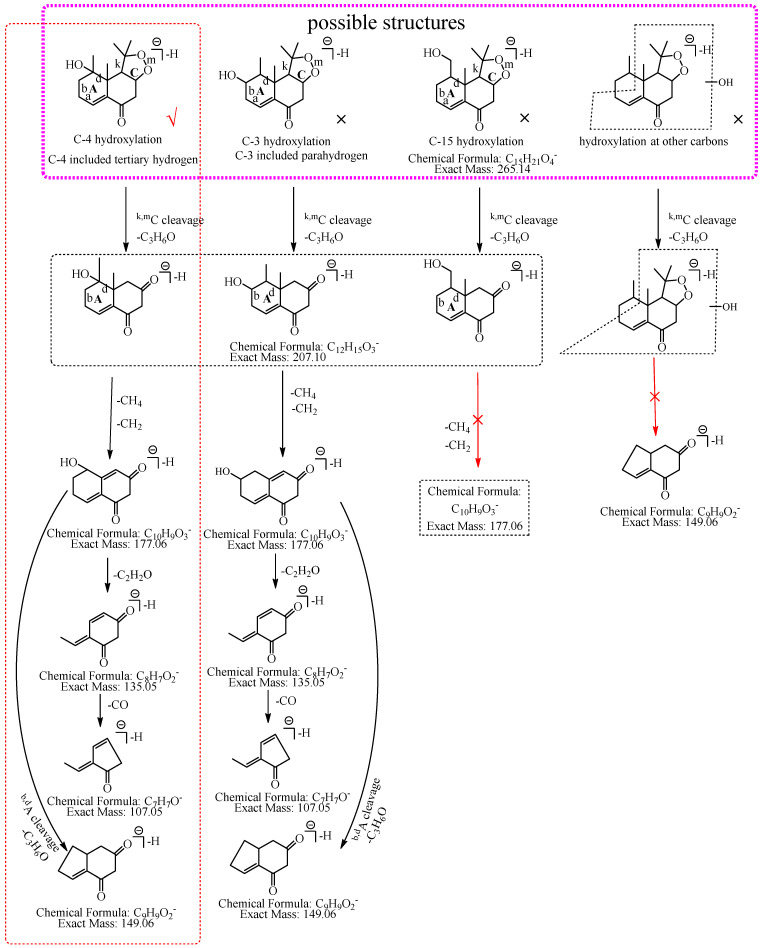
The proposed fragmentation pathways of M3 (4-hydroxyl nardosinone). “√” represents the possible structure. “×” represents the impossible structure. The purple rim represents the possible structures; A and C represent the hexatomic rings, respectively. The red rim represents the most possible fragmentation pathway.

M3: The ions at *m*/*z* 207.10 (C_12_H_15_O_3_) and *m*/*z* 149.06 (C_9_H_9_O_2_) were formed by the consecutive losses of two C_3_H_6_O from [M−H]^−^ in the MS^2^ spectra, which inferred that the hydroxylation occurred at C-3, C-4, or C-15. The ion at *m*/*z* 177.06 (C_10_H_9_O_3_) was formed by the consecutive losses of two 14.02 Da (CH_2_) from the ion at *m*/*z* 207.10, which could imply that hydroxylation did not occur at C-15. As described in the identification of M1, C-3 was a secondary carbon with parahydrogen, and C-4 was a tertiary carbon with tertiary hydrogen, which was more active than the parahydrogen of C-3, so it was inferred that the hydroxylation was more likely to occur at C-4. Therefore, M3 was preliminarily identified as 4-hydroxyl nardosinone. The possible structure and fragmentation pathways of M3 are shown in Figure 5.

**Figure 6:**
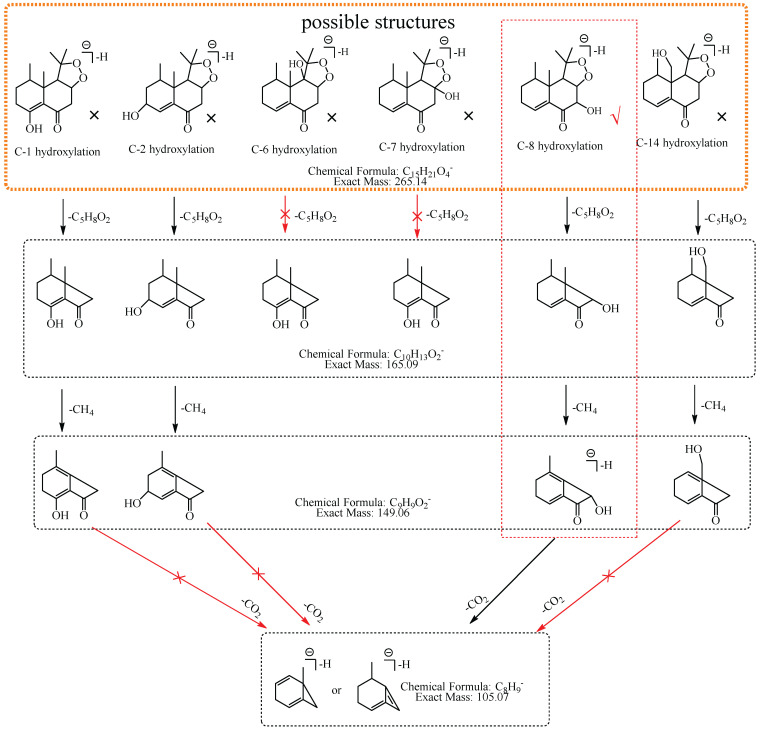
The proposed fragmentation pathways of M4 (8-hydroxyl nardosinone). “√” represents the possible structure. “×” represents the impossible structure. The orange rim represents the possible structures. The red rim represents the most possible fragmentation pathway.

M4: The [M−H]^−^ of M4 at *m*/*z* 265.14 directly lost C_5_H_8_O_2_ to produce *m*/*z* 165.09 (C_10_H_13_O_2_), while the ions at *m*/*z* 207.10 (C_12_H_15_O_3_) and *m*/*z* 193.09, which were respectively formed by the losses of 58.04 Da (C_3_H_6_O) and 72.06 Da (C_4_H_8_O) from [M−H]^−^_,_ were not observed in MS^2^ spectra, implying that the hydroxylation did not occur at C-2, C-3, C-4, C-6, C-7, C-12, C-13, or C-15. The ion at *m*/*z* 165.09 (C_10_H_13_O_2_) consecutively lost 16.03 Da (CH_4_) and 43.99 Da (CO_2_) to generate *m*/*z* 149.06 (C_9_H_9_O_2_) and *m*/*z* 105.07 (C_8_H_9_), suggesting that the hydroxylation did not occur at C-1, C-2, and C-14. Thus, it was speculated that the hydroxylation occurred at C-8, and M4 was preliminarily identified as 8-hydroxyl nardosinone. The possible structure and fragmentation pathways of M4 are shown in Figure 6.

**Figure 7:**
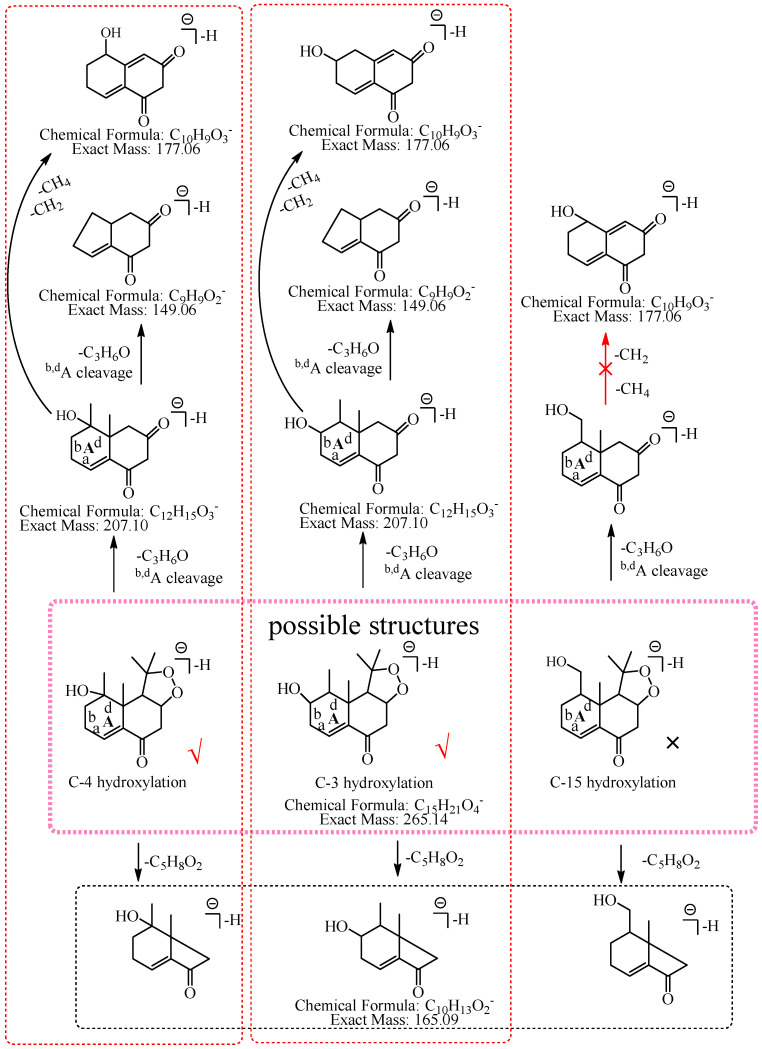
The proposed fragmentation pathways of M5 (3-hydroxyl nardosinone or 4-hydroxyl nardosinone). “√” represents the possible structure. “×” represents the impossible structure. The pink rim represents the possible structures; A represents the hexatomic ring. The red rims represent the most possible fragmentation pathways.

M5: The ions at *m*/*z* 207.10 (C_12_H_15_O_3_) and *m*/*z* 149.06 (C_9_H_9_O_2_) were formed by the consecutive losses of two C_3_H_6_O from [M−H]^−^ in the MS^2^ spectra, which inferred that the hydroxylation occurred at C-3, C-4, or C-15. The ions at *m*/*z* 191.07 (C_11_H_11_O_3_) and *m*/*z* 177.06 (C_10_H_9_O_3_) formed by the consecutive losses of 16.03 Da (CH_4_) and 14.02 Da (CH_2_) from the ion at *m*/*z* 207.10 were observed, which could infer that hydroxylation did not occur at C-15. If hydroxylation occurred at C-3, C-4, or C-15, the ion at *m*/*z* 207.10 (C_12_H_15_O_3_) would directly lose 70.04 Da (C_4_H_6_O) to form the ion at *m*/*z* 137.06 (C_8_H_9_O_2_). In addition, the ions at *m*/*z* 165.09 (C_10_H_13_O_2_) and *m*/*z* 137.06 (C_8_H_9_O_2_) formed by the consecutive losses of 42.01 Da (C_2_H_2_O) and 28.03 Da (C_2_H_4_) from the ion at *m*/*z* 207.10 (C_12_H_15_O_3_) were observed in the MS^2^ spectra. Therefore, M5 was preliminarily identified as 3-hydroxyl nardosinone or 4-hydroxyl nardosinone. The possible structure and fragmentation pathways of M5 are shown in Figure 7.

**Figure 8:**
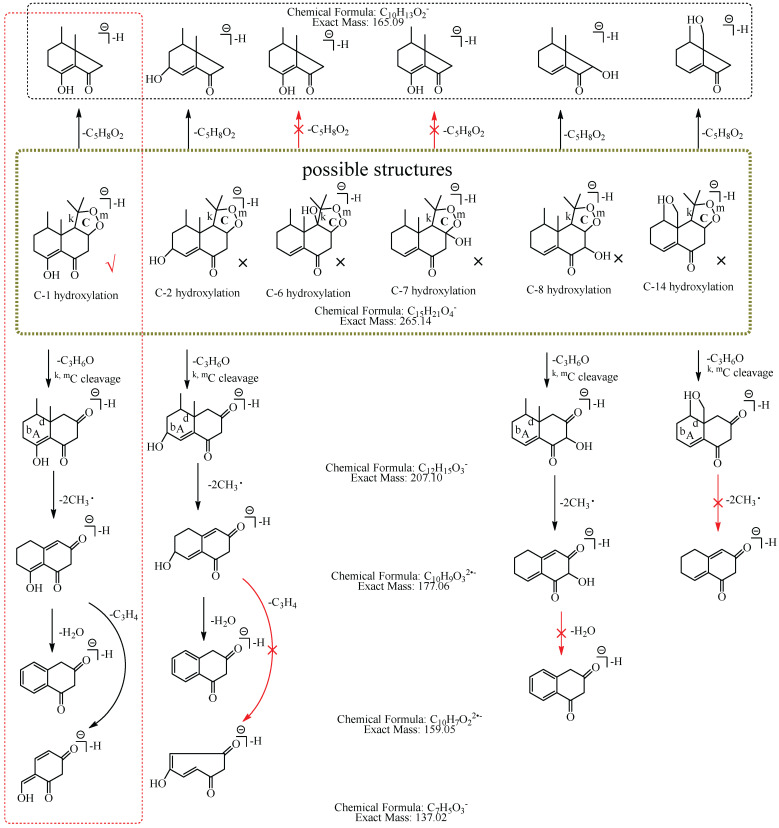
The proposed fragmentation pathways of M6 (1-hydroxyl nardosinone). “√” represents the possible structure. “×” represents the impossible structure. The olive rim represents the possible structures; C represents the hexatomic ring. The red rim represents the most possible fragmentation pathway.

M6: The [M−H]^−^ at *m*/*z* 265.14 of M6 lost 58.04 Da (C_3_H_6_O) to generate *m*/*z* 207 in MS^2^ spectra, while the ion at *m*/*z* 149.06, which was formed by the losses of two C_3_H_6_O from [M−H]^−^ was not observed, implying that the hydroxylation did not occur at C-3, C-4, C-12, C-13, or C-15. The ion *m*/*z* 265.14 ([M−H]^−^) lost 100.05 Da (C_5_H_8_O_2_) to generate *m*/*z* 165.09 (C_10_H_13_O_2_), indicating that the hydroxylation did not occur at C-6 and C-7. The characteristic fragment ion at *m*/*z* 177.06 (C_10_H_9_O_3_), which was formed by the losses of two 15.02 Da (CH_3_•) from *m*/*z* 207.10 (C_12_H_15_O_3_), was observed in MS^2^ spectra; indicating that the hydroxylation did not occur at C-14. The ion at *m*/*z* 177.06 (C_10_H_9_O_3_) lost 18.01 Da (H_2_O) to generate *m*/*z* 159.05 (C_10_H_7_O_2_), which implied that the hydroxylation did not occur at C-8. It also lost 40.03 Da (C_3_H_4_) to generate *m*/*z* 137.03 (C_7_H_5_O_3_), suggesting that the hydroxylation did not occur at C-2. Therefore, it was speculated that the hydroxylation should occur at C-1, and M6 was preliminarily identified as 1-hydroxyl nardosinone. The possible structure and fragmentation pathways of M6 are shown in Figure 8.

**Figure 9:**
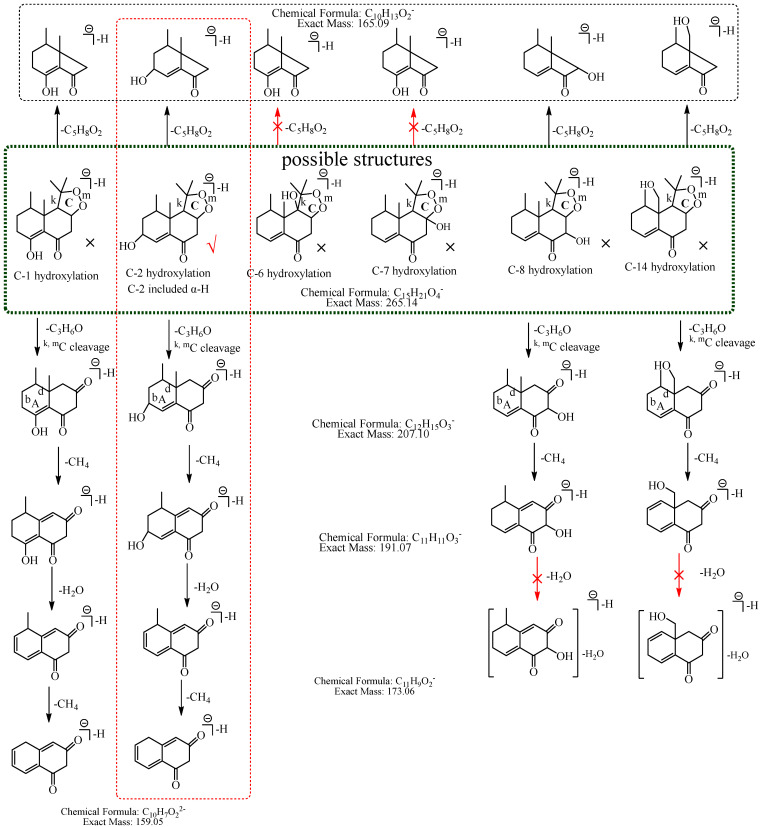
The proposed fragmentation pathways of M7 (2-hydroxyl nardosinone). “√” represents the possible structure. “×” represents the impossible structure. The atrovirens rim represents the possible structures; A and C represent the hexatomic rings. The red rim represents the most possible fragmentation pathway.

M7: In MS^2^ spectra, *m*/*z* 265.14 ([M−H]^−^) lost 58.04 Da (C_3_H_6_O) to generate *m*/*z* 207, while the ion at *m*/*z* 149.06, which was obtained by the losses of two C_3_H_6_O from [M−H]^−^ was not observed, implying that the hydroxylation did not occur at C-3, C-4, C-12, C-13 or C-15. The [M−H]^−^ could lose 100.05 Da (C_5_H_8_O_2_) to generate *m*/*z* 165.09 (C_10_H_13_O_2_), indicating that the hydroxylation did not occur at C-6 and C-7. *m*/*z* 191.07 (C_11_H_11_O_3_) lost 18.01 Da (H_2_O) to produce *m*/*z* 173.06 (C_11_H_9_O_2_), suggesting that the hydroxylation did not occur at C-8 and C-14. Therefore, the hydroxylation should occur at C-1 or C-2. C-2 has α-H of alkene, which is more active. Consequently, it was speculated that the hydroxylation more likely occurred at C-2, and M7 was identified as 2-hydroxyl nardosinone. The possible structure and fragmentation pathways of M7 are shown in Figure 9.

**Figure 10:**
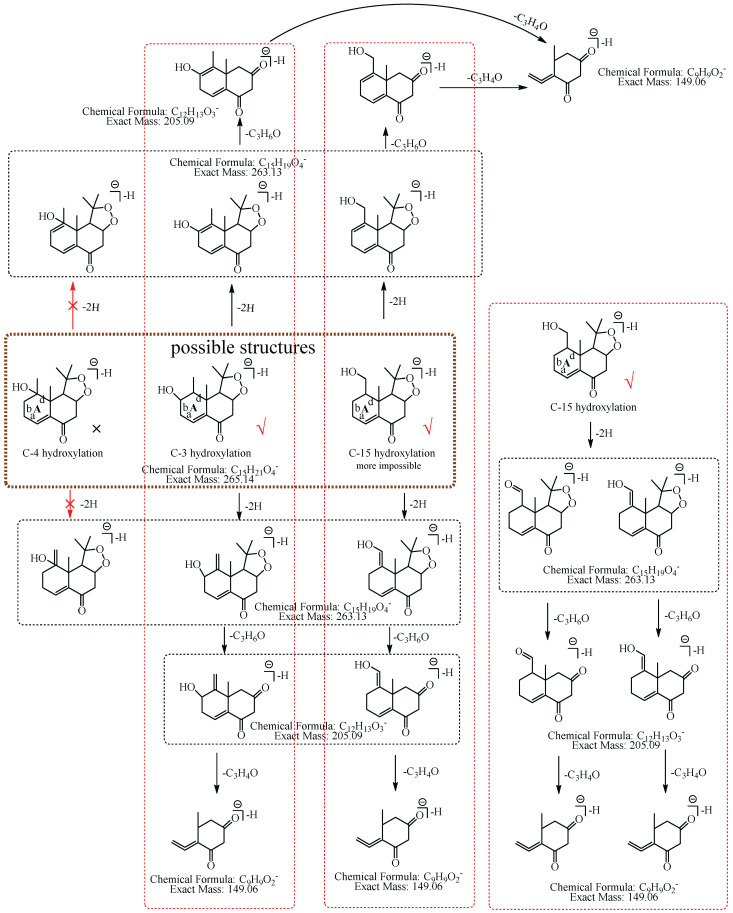
The proposed fragmentation pathways of M8 (3-hydroxyl nardosinone or 15-hydroxyl nardosinone). “√” represents the possible structure. “×” represents the impossible structure. The brown rim represents the possible structures; A represents the hexatomic ring. The red rims represent the most possible fragmentation pathways.

M8: The ions at *m*/*z* 205.09 (C_12_H_13_O_3_) and *m*/*z* 149.06 (C_9_H_9_O_2_) were formed by the consecutive losses of 58.04 Da (C_3_H_6_O) and 56.03 Da (C_3_H_4_O) from [M−H]^−^. Compared with the fragmentation characteristics of M1 (consecutive neutral losses of two C_3_H_6_O were observed), M8 did not directly lose the second C_3_H_6_O but lost C_3_H_4_O. It was speculated that the hydroxylation more likely occurred at C-3, C-4, or C-15 and a carbon–carbon double bond existed among C-3, C-4, and C-15. Therefore, the neutral loss of 56.03 Da (C_3_H_4_O) was speculated to be generated by the hydroxylation that occurred at C-15 and the dehydrogenation that occurred at C-3 and C-4, or the hydroxylation that occurred at C-15 and the dehydrogenation that occurred at C-15 and C-4, or the hydroxylation and dehydrogenation that both occurred at C-15, or the hydroxylation that occurred at C-3 and the dehydrogenation that occurred at C-3 and C-4. Therefore, M8 was preliminarily identified as 3-hydroxyl nardosinone or 15-hydroxyl nardosinone. The possible structure and fragmentation pathways of M8 are shown in Figure 10.

#### 2.2.2. Dihydroxylated Nardosinone and Their Glucuronides (M9−M13 and M14−M23)

M9−M13 showed [M−H]^−^ at *m*/*z* 281.14, and their molecular formulae were predicted as C_15_H_22_O_5_. The characteristic fragment ion at *m*/*z* 223.10 (C_12_H_15_O_4_) was formed by a loss of 58.04 Da (C_3_H_6_O) from [M−H]^−^ in the MS^2^ spectra. Compared to the nardosinone (C_15_H_22_O_3_), M9−M13 had two additional O atoms in their molecular formulae, thus, they were identified as dihydroxylated nardosinone isomers.

The molecular formulae of M14−M23 were verified to be C_21_H_30_O_11_ according to their [M−H]^−^ at *m*/*z* 457.17. In the MS^2^ spectra of M14, M15, M16, M19, and M20, *m*/*z* 281.14 (C_15_H_21_O_4_) formed by the loss of 176.03 Da (C_6_H_8_O_6_) from [M−H]^−^ was observed, and in the MS^2^ spectra of M17 and M23, the fragment ion at *m*/*z* 175.02 (C_6_H_7_O_6_) was detected. Therefore, M14, M15, M16, M17, M19, M20, and M23 were identified as dihydroxylated nardosinone glucuronides. The [M−H]^−^ consecutively lost 18.01 Da (H_2_O), 58.04 Da (C_3_H_6_O), and 176.03Da (C_6_H_8_O_6_) to produce *m*/*z* 439.16 (C_21_H_27_O_10_), *m*/*z* 381.12 (C_18_H_21_O_9_), and *m*/*z* 205.09 (C_12_H_13_O_3_) in MS^2^ spectra of M22; the ion at *m*/*z* 205.09 (C_12_H_13_O_3_) formed by the loss of 176.03Da (C_6_H_8_O_6_) from [M−H]^−^ was observed in MS^2^ spectra of M18 and M21. M18, M21, and M22, as the isomers of M14, M15, M16, M17, M19, M20, and M23, were identified as dihydroxylated nardosinone glucuronide isomers.

#### 2.2.3. Metabolites Formed by Hydroxylation, and Dehydrogenataion or Dehydration of Nardosinone

M24 showed [M−H]^−^ at *m*/*z* 247.13, and its molecular formula was predicted as C_15_H_20_O_3_. [M−H]^−^ sequentially lost two C_3_H_6_O to generate *m*/*z* 189.09 (C_12_H_13_O_3_) and *m*/*z* 131.05 (C_9_H_7_O_2_), implying that the hydroxylation should occur at C-3, C-4 or C-15. Moreover, M24 had one fewer O atom and two fewer H atoms in its molecular formula in comparison with the M1−M8; thus, it was identified as a hydroxylated dehydrated nardosinone isomer.

M25−M30 had the molecular formulae of C_15_H_20_O_4_, which was predicted by their [M−H]^−^ at *m*/*z* 263.13. The characteristic fragment ion at *m*/*z* 245.11 (C_15_H_18_O_3_) was formed by the loss of 18.02 Da (H_2_O) from [M−H]^−^ in MS^2^ spectra of M25 and M26; the ion at *m*/*z* 205.09 (C_12_H_13_O_3_) was formed by the loss of 58.04 Da (C_3_H_6_O) from [M−H]^−^ in MS^2^ spectra of M27−M30. Compared to the M1−M8, M25−M30 had two fewer H atoms in their molecular formulae, and the characteristic fragment ion at *m*/*z* 263.13 was observed in the MS^2^ spectra of M8. Thus, they were identified as hydroxylated dehydrogenated nardosinone isomers.

Dihydroxylated dehydrogenated nardosinone and their phase II metabolites (M31−M37 and M38−M40): M31−M37 showed [M−H]^−^ at *m*/*z* 279.12, and their molecular formulae were predicted as C_15_H_20_O_5_. [M−H]^−^ lost 58.04 Da (C_3_H_6_O) to produce *m*/*z* 221.08 (C_12_H_13_O_4_) in MS^2^ spectra of M31, M32, M35, and M37. M31−M37 had one more O atom than M25−M30; therefore, they were identified as dihydroxylated dehydrogenated nardosinone isomers. M38−M40 showed [M−H]^−^ at *m*/*z* 455.16, and their molecular formulae were predicted as C_21_H_28_O_11_. The ion at *m*/*z* 279.13 (C_15_H_19_O_5_) was formed by the loss of 176.03 Da (C_6_H_8_O_6_) from [M−H]^−^ in MS^2^ spectra of M38 and M39, and *m*/*z* 175.02 (C_6_H_7_O_6_) was observed in the MS^2^ spectra of M40. Thus, M38−M40 were speculated as dihydroxylated dehydrogenated nardosinone glucuronide isomers.

The molecular formula of M41 was predicted to be C_15_H_18_O_4_ based on its [M−H]^−^ at *m*/*z* 261.11. [M−H]^−^ lost 58.04 Da (C_3_H_6_O) to generate *m*/*z* 203.07 (C_12_H_11_O_3_). Compared to the M31−M37, M41 had one fewer O atom and two fewer H atoms in its molecular formula. Thus, M41 was speculated as dihydroxylated dehydrogenated dehydrated nardosinone.

M42−M44 showed [M−H]^−^ at *m*/*z* 295.12, and their molecular formulae were predicted as C_15_H_20_O_6_. The characteristic fragment ions at *m*/*z* 277.11 (C_15_H_19_O_6_), *m*/*z* 233.12 (C_14_H_19_O_4_), and *m*/*z* 205.13 (C_13_H_19_O_3_) were formed by consecutive losses of 18.01 Da (H_2_O), 43.99 Da (CO_2_), and 27.99 Da (CO) from [M−H]^−^ in MS^2^ spectra of M43. Compared to M31−M37, M42−M44 had one more O atom in their molecular formulae. Thus, M42−M44 was speculated as trihydroxylated dehydrogenated nardosinone isomers.

#### 2.2.4. Metabolites Formed by Hydroxylation, Hydrogenation, and Carboxylation of Nardosinone

The molecular formulae of M45−M48 were predicted to be C_16_H_24_O_6_ based on their [M−H]^−^ at *m*/*z* 311.15. [M−H]^−^ lost 43.99 Da (CO_2_) to generate *m*/*z* 267.16 (C_15_H_24_O_4_) in the MS^2^ spectra of M47, and it had one more O atom and two more H atoms compared with nardosinone; thus, M47 was speculated as hydrogenated hydroxylated carboxylated nardosinone. M45, M46, and M48 were the isomers of M47 and were identified as hydrogenated hydroxylated carboxylated nardosinone isomers.

#### 2.2.5. Nardosinone Glucuronides

M49−M53 showed [M−H]^−^ at *m*/*z* 425.18, and their molecular formulae were predicted as C_21_H_30_O_9_. [M−H]^−^ lost 250.16 Da (C_15_H_22_O_3_, nardosinone) to generate *m*/*z* 175.02 (C_6_H_7_O_6_) in MS^2^ spectra of M49−M52. Therefore, M49−M53 were identified as nardosinone glucuronides. The characteristic fragment ion at *m*/*z* 367.14 (C_18_H_23_O_8_) was formed by the loss of 58.04Da (C_3_H_6_O) from [M−H]^−^ in the MS^2^ spectra of M50 and M51, suggesting that the glucuronidation did not occur at the hydroxyl, which was generated by the cleavage of the peroxide bridge, but probably occurred at the hydroxyl of C-7 or C-9. While the neutral loss of 58.04 Da (C_3_H_6_O) in MS^2^ spectra of M49, M52, and M53 was not observed, suggesting that the glucuronidation probably occurred at the hydroxyl, which was generated by the cleavage of the peroxide bridge.

The molecular formulae of M54−M56 were verified to be C_21_H_32_O_10_ based on their [M−H]^−^ at *m*/*z* 443.19. M54−M56 had one more O atom and two more H atoms than M49−M53; therefore, they were identified as hydrogenated hydroxylated nardosinone glucuronides. In MS^2^ spectra of M55, [M−H]^−^ consecutively lost 58.04 Da (C_3_H_6_O) and 18.01 Da (H_2_O) to generate *m*/*z* 385.15 (C_18_H_25_O_9_) and *m*/*z* 367.14 (C_18_H_23_O_8_), and *m*/*z* 385.15 (C_18_H_25_O_9_) further lost 44.03 Da (C_2_H_4_O) to generate *m*/*z* 341.12 (C_16_H_21_O_8_). These implied that it was impossible that hydroxylation occurred at C-1, C-2, C-6, C-8, and C-14 and glucuronidation occurred at the hydroxyl of C-7, and hydroxylation, hydrogenation, and glucuronidation simultaneously occurred at C-7 of M55. In addition, *m*/*z* 193.03 (C_6_H_9_O_7_, glucuronic acid ion) was also observed in MS^2^ of M55, and it was speculated that the compound easily lost glucuronic acid and formed conjugated systems in the aglycone, and the possibility that hydroxylation and glucuronidation simultaneously occurred at C-4, C-14, and C-15 was basically excluded because it could not form conjugated systems after losing glucuronic acid of M55. Moreover, the possibility that hydroxylation and glucuronidation simultaneously occurred at C-1 was also excluded, owing to the glycosidic bond of enol form hydroxyl at C-1 being difficult to break. In conclusion, it was speculated that M55 was generated by the hydrogenation of the peroxide bridge and simultaneous hydroxylation and glucuronidation at C-2, C-3, C-6, or C-8.

### 2.3. Identification of Metabolites with the Skeleton of Nardosinone Acid

#### 2.3.1. Metabolites Formed by Dihydroxylation, Dehydration, and Glucuronidation of Nardosinone Acid

M57 had the molecular formula of C_12_H_16_O_4_ predicted by its [M−H]^−^ at *m*/*z* 223.10. The characteristic fragment ions at *m*/*z* 191.11 (C_12_H_15_O_2_) and *m*/*z* 161.10 (C_11_H_13_O) were formed by the consecutive losses of 31.99 Da (2O, didehydroxylation) and 30.01 Da (CH_2_O) from [M−H]^−^. M57 had two additional O atoms in its molecular formula by comparison with nardosinone. Therefore, it was speculated as dihydroxylated nardosinone acid.

C_12_H_14_O_3_ and its phase II metabolites (M58−M61 and M62). M58−M61 showed [M−H]^−^ at *m*/*z* 205.09, and their molecular formulae were predicted as C_12_H_14_O_3_. [M−H]^−^ continuously lost two CH_3_• to generate *m*/*z* 190.07 (C_11_H_10_O_3_•) and *m*/*z* 175.05 (C_10_H_7_O_3_) in MS^2^ spectra of M58−M61. M58−M61 had one fewer O atom and two fewer H atoms than M57. Therefore, they were speculated as dehydrated dihydroxylated nardosinone acid isomers. M62 had the molecular formula of C_18_H_22_O_9_ predicted by its [M−H]^−^ at *m*/*z* 381.12. The [M−H]^−^ of M62 lost 176.03 Da (C_6_H_8_O_6_) to produce *m*/*z* 205.09 (C_12_H_13_O_3_), and the ions at *m*/*z* 190.06 and *m*/*z* 175.04 were identical with those of M58−M61. Therefore, M62 was identified as a dehydrated double hydroxylated nardosinone acid glucuronide.

#### 2.3.2. Glucuronides and Sulfates of Nardosinone Acid

M63−M65 had the molecular formulae of C_18_H_24_O_8_ predicted by their [M−H]^−^ at *m*/*z* 367.14. In their MS^2^ spectra, [M−H]^−^ lost 176.03 Da (C_6_H_8_O_6_) to produce the *m*/*z* 191.11 (C_12_H_15_O_2_). Therefore, they were identified as nardosinone acid glucuronide isomers. The ion at *m*/*z* 193.04 (C_6_H_9_O_7_) was observed in the MS^2^ spectra of M63−M64, implying that the glycosidic bond broke easily and there were no conjugated systems near the glycosidic bond. Because enol form hydroxyl of C-7 and carbonyl of C-9 both had a stable conjugated system, glucuronidation should occur at hydroxyl of C-6 (the rearrangement of C-7 hydroxyl to C-6) for M63−M64. The ion at *m*/*z* 193.04 was not observed in the MS^2^ spectra of M65. Considering the energy difference required for bond cleavage, it was speculated that the glucuronidation occurred at the enol form hydroxyl of C-7 or the carbonyl of C-9, but the carbonyl of C-9 has already formed a stable conjugated system with the carbon–carbon double bond between C-10 and C-1, so the possibility that glucuronidation occurred at the carbonyl of C-9 was less. Therefore, it was speculated that the glucuronidation occurred at the enol form hydroxyl of C-7 in M65.

M66−M70 showed [M−H]^−^ at *m*/*z* 271.06, and their molecular formulae were predicted as C_12_H_16_O_5_S. In the MS^2^ spectra of M66, its [M−H]^−^ lost 79.96 Da (SO_3_) to produce *m*/*z* 191.11 (C_12_H_15_O_2_), while the ions at *m*/*z* 96.96 (HSO_4_) and *m*/*z* 80.96(HSO_3_) were not observed. Considering the energy difference required for bond cleavage, it was speculated that the sulfation occurred at enol form hydroxyl. However, the carbonyl of C-9 already formed a stable conjugated system with the carbon–carbon double bond between C-10 and C-1, so the possibility that sulfation occurred at the carbonyl of C-9 was less. Therefore, it was speculated that the sulfation occurred at the enol hydroxyl of C-7 in M66. In the MS^2^ spectra of M67, its [M−H]^−^ lost 81.97 Da (H_2_SO_3_) to produce *m*/*z* 189.10 (C_12_H_13_O_2_); the ion at *m*/*z* 80.96 (HSO_3_) was observed in the MS^2^ spectra of M69; the ions at *m*/*z* 96.96 (HSO_4_) and *m*/*z* 80.96 (HSO_3_) were both observed in the MS^2^ spectra of M68 and M70. The sulfate ion at *m*/*z* 96.96 (HSO_4_), the sulfonyl ion at *m*/*z* 80.96 (HSO_3_), and the neutral loss of 81.97 Da (H_2_SO_3_) all indicated that the sulfate ester and the vicinal H were simultaneously lost, suggesting that the hydroxyl, which was sulfated was not enol form hydroxyl and did not in the conjugated system. Thus, it was speculated that the sulfation should occur at the hydroxyl of C-6. Therefore, M66−M70 were identified as nardosinone acid sulfates and the possible structures and the fragmentation pathways are shown in Figure S4.

The molecular formulae of M71−M76 were verified to be C_12_H_18_O_5_S based on their [M−H]^−^ at *m*/*z* 273.08. The characteristic fragment ions at *m*/*z* 191.11 (C_12_H_15_O_2_) and *m*/*z* 80.96 (HSO_3_) were observed in the MS^2^ spectra of M73, M74, and M76, and the characteristic fragment ion at *m*/*z* 80.96 (HSO_3_) was observed in the MS^2^ spectra of M71, M72, and M75. As mentioned above, it was speculated that the sulfation did not occur at the enol form hydroxyl of M71−M76, and the possible sulfation sites were deduced as follows: (1) if sulfation occurred at hydroxyl of C-6 and the hydrogenation occurred at carbon–carbon double bond between C-7 and C-8, the conjugated system could not be formed after losing H_2_SO_3_, thus, it was less likely to occur; (2) if sulfation occurred at hydroxyl of C-6 and the hydrogenation occurred at carbon–carbon double bond between C-10 and C-1, the conjugated system could be formed after losing H_2_SO_3_, thus, it was more likely to occur; (3) if sulfation occurred at hydroxyl of C-6 and the hydrogenation occurred at carbonyl of C-9, the conjugated system could not be formed after losing H_2_SO_3_, thus, it was less likely to occur; (4) if sulfation occurred at hydroxyl of C-7 (reduction of carbonyl), the conjugated system could be formed after losing H_2_SO_3_, thus, it was more likely to occur; (5) if the sulfation occurred at hydroxyl of C-9 (reduction of carbonyl), the conjugated system could be formed with the enol hydroxyl of C-7 after losing H_2_SO_3_, but the carbonyl of C-9 was less likely to be hydrogenated because it already formed a conjugated system, therefore, the possibility that sulfation and hydrogenation simultaneously occurred at carbonyl of C-9 was less. Since the sulfation could not occur at the enol form hydroxyl (because no conjugated system could be formed after losing H_2_SO_3_) for M71−M76, when the hydrogenation occurred at the carbon–carbon double bond between C-10 and C-1, the sulfation should not occur at the enol form hydroxyl of C-7 and C-9. In conclusion, it was speculated that the sulfation should occur at the hydroxyl of C-6 and the hydrogenation occur at the carbon–carbon double bond between C-10 and C-1, or that the sulfation and hydrogenation simultaneously occur at the enol form hydroxyl of C-7. Compared to M66−M70, M71−M76 had two additional H atoms in their molecular formulae, therefore, M71−M76 were identified as hydrogenated nardosinone acid sulfates, and their possible structures and fragmentation pathways are shown in Figure S5.

### 2.4. Identification of Several Metabolites of Nardosinone with the Skeleton of Nardosinone Acid in the Fast Validation Experiment

**Figure 11:**
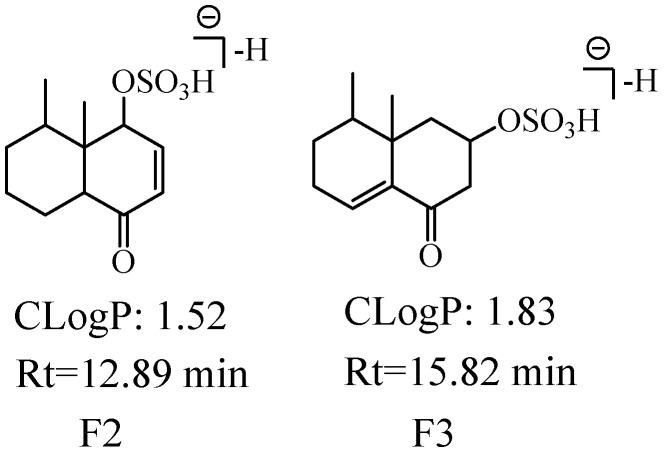
The possible structures of F2 and F3. The CLogP represents CLogP values, Rt represents retention time.

We detected and identified three metabolites (F1-F3, the extracted ion chromatogram of them is shown in Figure S6). The molecular formulae of F1-F3 were predicted to be C_12_H_18_O_5_S according to their [M−H]^−^ at *m*/*z* 273.08. The ion at *m*/*z* 79.96 (SO_3_) was observed in the MS^2^ spectra of F1, implying that the sulfation occurred at the enol form hydroxyl (C-7 or C-9); the ion at *m*/*z* 80.96 Da (HSO_3_) was observed in the MS^2^ spectra of F2 and F3, indicating that the sulfation occurred at the hydroxyl of C-6 or C-7 (reduction of carbonyl). F1-F3 showed [Aglycon−H]^−^ (C_12_H_17_O_2_) at *m*/*z* 193.12, which had two more H atoms than the molecular formula of nardosinone acid, thus F1-F3 were speculated as hydrogenated nardosinone acid sulfates. The larger CLogP value means a longer retention time in reversed-phase UHPLC, thus, the possible structures of F2 (ClogP = 1.52 and t_R_ = 12.89 min) and F3 (ClogP = 1.78 and t_R_ = 15.82 min) are shown in Figure 11.

## 3. Discussion

### 3.1. Comparative Analysis of the Metabolic Characteristics of Nardosinone and Other Sesquiterpene Peroxides

Artemisinin compounds are typical representatives of sesquiterpene peroxides, and many researchers have carried out the metabolism studies of them owing to their unique structures and antimalarial activity. It was inferred that the peroxide bridge was broken into a mono-oxygen bridge based on the metabolic researches of dihydroartemisinininin derived-dimer and dihydroartemisinin. Moreover, the main metabolic reactions included hydroxylation, dehydration, glucuronidation, carbonylation, dehydrogenation, etc. It was worth noting that the metabolic reactions of mono-hydroxylation, dihydroxylation, trihydroxylation, and quahydroxylation were not found in the metabolism of dihydroartemisinin but dihydroartemisinininin derived-dimer. Furthermore, carboxylated metabolites were found in the in vitro metabolism of artemether [31,32]. The in vivo and in vitro metabolism of artemisinin and dihydroartemisinin manifested that the peroxide bridge could be broken into a mono-oxygen bridge and the metabolic reactions mainly included dihydroxylation, deoxidation, hydroxylation, and glucuronidation, and 25 metabolites of artemisinin and 16 metabolites of dihydroartemisinin were identified, respectively [25]. The metabolites of hydroxylated dihydroartemisinin (DHA+O), dehydro-hydroxylated dihydroartemisinin (DHA−H2+O), and dehydration-hydroxylated dihydroartemisinin (DHA−H_2_O+O) were identified in the in vivo metabolism of dihydroartemisinin [33], which resembled the metabolites of hydroxylated nardosinone, dehydro-hydroxylated nardosinone, and dehydra-hydroxylated nardosinone in this study. Compared with the metabolism of other sesquiterpene peroxides, combined with the metabolic pathway and metabolites of nardosinone, we speculated that the peroxide bridge cleavage (loss of C_3_H_6_O) of nardosinone was more likely to generate enol hydroxyl or carbonyl other than the mono-oxygen bridge, which was inconsistent with the cleavage of the peroxide bridge and the formation of a mono-oxygen bridge in the metabolism of artemisinin. Furthermore, the common metabolic reactions reported in other sesquiterpene peroxides were also discovered in the metabolism of nardosinone, e.g., hydroxylation, carboxylation, sulfation, glucuronidation, etc.

### 3.2. Discussion on the Origin of Metabolites with the Nardosinone Acid Skeleton

It was reasonable that the hydroxy-isopropyl (C_3_H_6_O) could be lost in the fragmentation pathway of nardosinone, while we found many metabolites with the nardosinone acid skeleton (e.g., M57−M76) in biological samples. The loss of hydroxy-isopropyl involved the cleavage of the C-C bond, which is a rare metabolic reaction. Therefore, we designed a fast validation experiment to confirm it. If metabolites with a nardosinone acid skeleton are detected in this experiment, it means that they are indeed generated by in vivo metabolism.

According to the literature, nardosinone is stable under the conditions of an alkaline, low temperature, and away from light [18]. Therefore, the environmental factors should be strictly controlled to inhibit the degradation of nardosinone during the experimental processes.

In our fast validation experiment, nardosinone was suspended in a 0.5% carboxymethyl cellulose sodium (CMC-Na) solution (alkaline) and stored at −20 °C in a dark place. Then the mice were orally administered with nardosinone once, and in the first-hour urine samples of mice were collected and immediately filtered through a 0.22-μm membrane and analyzed by using UHPLC-Q-TOF-MS as quickly as possible. However, the metabolites with a nardosinone acid skeleton were still detected, which indicated that nardosinone acid and its metabolites were not generated by experimental operations such as ultrasonic extraction but generated by the in vivo metabolism of nardosinone. Furthermore, the results of the liver metabolism of the *Cistanche deserticola* total glycosides showed that the *Cistanche deserticola* total glycosides could lose the C_3_H_5_O group (*m*/*z* 57.06) to generate the metabolite methylated laricin [34]. Therefore, we consider that it is reasonable that nardosinone can lose hydroxy-isopropyl (C_3_H_6_O) to generate nardosinone acid in the in vivo metabolism.

But we still do not know where nardosinone loses the hydroxy-isopropyl (C_3_H_6_O) to generate nardosinone acid in vivo, and it could be the stomach, the liver, the intestinal tract, or somewhere else. We speculate that nardosinone is transformed into nardosinone acid by the intestinal microflora. Therefore, it deserves further research to explore the in vivo formation mechanism of nardosinone acid.

## 4. Materials and Methods

### 4.1. Reagent

The purity of nardosinone was greater than 98% (UHPLC, 254 nm) and it was purchased from Chengdu Push Bio-technology (Chengdu, China, Lot No. PS010660). HPLC-grade formic acid (Lot No. 212271), UHPLC-grade acetonitrile (Lot No. 207296), and HPLC-grade methanol (Lot No. 203511) were purchased from Thermo Fisher Scientific (Waltham, MA, USA); HPLC-grade ethanol (Lot No. 32061) was purchased from Beijing Tong Guang Fine Chemicals Company (Beijing, China). Sodium carboxymethyl cellulose (Lot No. A18105) was purchased from Sinopharm Chemical Reagents Co., Ltd. (Shanghai, China). Ultrapure water was prepared using a Milli-Q Integral 3 ultrapure water machine (Millipore, Billerica, MA, USA).

### 4.2. Animal Experiments

Nine ICR mice (male, 30 ± 2 g) were purchased from the Department of Laboratory Animal Sciences at the Peking University Health Science Center. They were randomized into three groups (test group I, test group II, and blank group) with three mice in each group. The experiment lasted for 10 d for the test group I and blank group, and 4 d for group II. All mice were housed in mouse metabolism cages with water and food ad libitum for the first three days, then the mice were dosed by gavage once per day for the following 7 d for the test group I and blank group, and 1 d for the test group II. The mice of test groups were orally administered with a dose of 80 mg/kg mouse-weight nardosinone suspended in 2.0 mL 0.5% carboxymethyl cellulose sodium (CMC-Na) solution, and the mice of blank group were orally administered with the same volume of 0.5% carboxymethyl cellulose sodium (CMC-Na) solution. The mice were allowed to eat and drink ad libitum. All animal experiments were approved by the Animal Ethics Committee of Peking University Health Science Center (approval number: LA2019117).

### 4.3. Samples Collection and Preparation

#### 4.3.1. Collection of Samples

In total, 8 mL ethanol was added into each urine collection tube for bacteriostasis before each urine collection. All the urine and feces were collected during the six days of dosing for the test group I and blank group, and one hour after the last oral administration, the blood was collected into 1.5 mL heparin sodium-containing tubes by excising the eyeballs. All samples were kept at −80 °C.

The first-hour urine of mice after orally administered with nardosinone was collected for the test group II, and was immediately filtered through 0.22-μm membrane, and analyzed by UHPLC-Q-TOF-MS directly. It took 2.5 h from drug administration to obtain mass spectra data.

#### 4.3.2. Preparation of Samples

All urine samples from the test group I and blank group were merged into two samples (test group I and blank group), respectively. Each sample was centrifuged at 8000 rpm at 4 °C for 15 min, and the supernatant was harvested, concentrated, and dried at 55 °C. Then, the urine residue was ultrasonically extracted with 10 times volume of methanol for 30 min and filtered to get the supernatant. The supernatant was evaporated to dryness at 55 °C. Finally, 0.5 g residue was dissolved in 1.0 mL methanol, and was filtered through 0.22-μm membrane, and stored at −80 °C before further analysis.

All feces samples from the test group I and blank group were combined into two samples (test group I and blank group) and were dried at 50 °C for 48 h and mashed. Each feces sample was ultrasonically extracted 30 min with 10 times volume of methanol for three times. The three extracts were filtered and merged, and dried at 55 °C. Next, the residue was ultrasonically extracted by 10 times volume of methanol for 30 min and centrifuged at 8000 rpm at 4 °C for 15 min to get the supernatant, which was further evaporated to dryness at 55 °C to obtain the residue. Finally, 0.5 g residue was dissolved in 1.5 mL methanol, and was filtered through 0.22-μm membrane, and stored at −80 °C before further analysis.

All plasma samples from the test group I and blank group were mixed into two samples (test group I and blank group) and centrifuged at 5000 rpm at 4 °C for 15 min to collect 1.0 mL supernatant, and it was mixed with 5 mL methanol and centrifuged at 5000 rpm at 4 °C for 15 min. The supernatant was separated and dried by nitrogen blow at 40 °C. Finally, 0.2 mg residue was dissolved in 0.3 mL methanol, and filtered through 0.22-μm membrane, and stored at −80 °C before further analysis.

### 4.4. Instrumentation and Analytical Conditions

Ultra-performance liquid chromatography–quadrupole time-of-flight mass spectrometry (UHPLC-Q-TOF-MS) analysis was performed on the SCIEX Triple TOF 6600 and SCIEX Exion LC AD System (UHPLC). All data were analyzed by PeakView v.1.2 software. Chromatographic separations were performed on an ACQUITY UPLC BEH C18 column (2.1 × 150 mm, 1.7 μm, Waters, USA) with a VanGuard Pre-Column (2.1 × 5 mm, 1.7 μm, Waters, USA). The column temperature was 35 °C; the injection volume was 2 μL, and the flow rate was 0.3 mL/min. The mobile phase was 0.1% aqueous solution of formic acid (A) and acetonitrile (B). The gradient elution program was set as follows: 0.01−3 min, 0.5% B; 3−6 min, 0.5%−8% B; 6−20 min, 8%−15% B; 20−33 min, 15%−60% B; 33−35 min, 60%−100% B; 35−38 min, 100% B; 38−39 min, 100%−0.5% B; 39−43 min, 0.5% B. The MS parameters were the following: electrospray ionization, negative ion (ESI) mode; full-scan mass spectra, *m*/*z* 100−1000 (MS) and *m*/*z* 50−1000 (MS^2^); Gas1, 60 psi; Gas2, 60 psi; Curtain gas, 35 psi; source temperature, 600 °C; Ion spray voltage, −4500 V (negative ion); Declustering potential, 60/−60 V; Collision energy, 35 ± 15 V.

### 4.5. Identification of the Existence Forms of Nardosinone In Vivo (Original Constituents and Metabolites)

The base peak chromatograms (BPCs) of the drug-containing group and blank group samples were compared to find the distinguishing peaks and tentatively determine the in vivo existence forms of nardosinone. Afterward, the distinguishing peaks were confirmed by comparing the corresponding extracted ion chromatograms (EICs) of the drug-containing group and blank group. If a compound could be detected by the extracted ion chromatogram (EIC) of the drug-containing group but did not exist in blank group, it was preliminarily verified as a metabolite. Furthermore, the MS data of reference substances, the MS fragmentation information reported in the literature, and the information obtained by searching the SciFinder database could all be used to identify the in vivo forms [35,36].

## 5. Conclusions

The in vivo metabolism of nardosinone was studied for the first time in mice. A total of 76 new metabolites were identified by UHPLC-Q-TOF-MS technology, and the metabolic reactions mainly included hydroxylation, dehydration, hydrogenation, sulfation, glucuronidation, demethylation, carboxylation, etc. There were 56 and 20 metabolites with the skeletons of nardosinone and nardosinone acid, respectively. It was confirmed that nardosinone could be biotransformed into nardosinone acid or isomer in vivo based on the results of the fast validation experiment. These results will be conducive to an in-depth study of the in vivo effective forms of nardosinone and NRR and will have certain reference values for the in vivo metabolism studies of other sesquiterpene peroxides.

## Figures and Tables

**Table 1:** Identification of nardosinone and its 76 metabolites in mice urine, feces, and plasma samples by UHPLC-Q-TOF-MS.

No	tR ^1^ (min)	Formula	Major Negative Fragmentions	Meas. ^1^ (Da)	Pred. ^1^ (Da)	Error (ppm)	Reaction	U ^1^	P ^1^	F ^1^
M0	27.41	C_15_H_22_O_3_	191.1085, 175.0770, 161.0614, 149.0610, 105.0696	249.1497	249.1496	0.3	nardosinone ^2^	+ ^1^	+	− ^1^
M1	11.90	C_15_H_22_O_4_	207.1027, 191.0711, 177.0558, 159.0453, 149.0608, 105.0709	265.1443	265.1445	−0.9	+O isomer 1	−	+	−
M2	14.14	C_15_H_22_O_4_	193.0872, 165.0980, 149.0620, 105.0699	265.1438	265.1445	−2.8	+O isomer 2	−	+	−
M3	10.91	C_15_H_22_O_4_	207.1026, 177.0556, 159.0438, 149.0601, 135.0466, 107.0509, 105.0703	265.1442	265.1445	−1.3	+O isomer 3	+	−	−
M4	13.39	C_15_H_22_O_4_	165.0920, 149.0595, 105.0723	265.1442	265.1445	−1.3	+O isomer 4	+	+	−
M5	18.09	C_15_H_22_O_4_	207.1037, 191.0708, 177.0530, 175.0761, 173.0626, 165.0922, 159.0445, 149.0598, 137.0605	265.1439	265.1445	−2.4	+O isomer 5	+	−	−
M6	22.14	C_15_H_22_O_4_	207.1027, 177.0584, 165.0929, 159.0418, 137.0617, 137.0262	265.1442	265.1445	−1.3	+O isomer 6	+	−	−
M7	23.88	C_15_H_22_O_4_	207.1030, 191.0725, 173.0624, 165.0937, 159.0452, 137.0590	265.1442	265.1445	−1.3	+O isomer 7	+	+	−
M8	26.34	C_15_H_22_O_4_	263.1324, 205.0887, 191.0714, 149.0589	265.1446	265.1445	0.3	+O isomer 8	+	+	−
M9	11.33	C_15_H_22_O_5_	223.1047, 209.0841, 147.0411	281.1395	281.1394	0.2	+2O isomer 1	+	−	−
M10	16.28	C_15_H_22_O_5_	223.0588, 205.0879, 190.0647, 175.0414	281.1392	281.1394	−0.9	+2O isomer 2	+	−	−
M11	12.24	C_15_H_22_O_5_	223.0962, 209.0813, 205.0852	281.1396	281.1394	0.5	+2O isomer 3	−	+	−
M12	15.94	C_15_H_22_O_5_	223.1044, 165.0567, 137.0594	281.1388	281.1394	−2.3	+2O isomer 4	−	+	−
M13	20.30	C_15_H_22_O_5_	223.0971, 209.0801	281.1399	281.1388	1.6	+2O isomer 5	−	+	−
M14	8.84	C_21_H_30_O_11_	439.1678, 281.1111, 175.0257	457.1717	457.1715	0.4	+2O, +GlcUA	+	−	−
M15	9.91	C_21_H_30_O_11_	281.1376, 175.0245	457.1706	457.1715	−2.0	+2O, +GlcUA	+	−	−
M16	17.64	C_21_H_30_O_11_	439.1652, 281.1021, 263.1320, 205.0887	457.1699	457.1715	−3.6	+2O, +GlcUA	+	−	−
M17	8.20	C_21_H_30_O_11_	205.0872, 175.0286	457.1701	457.1715	−3.1	+2O, +GlcUA	+	−	−
M18	9.45	C_21_H_30_O_11_	381.1170, 205.0859	457.1718	457.1715	0.6	+2O, +GlcUA isomer 1	−	+	−
M19	10.52	C_21_H_30_O_11_	281.1419, 205.0972	457.1709	457.1715	−1.4	+2O, +GlcUA	−	+	−
M20	13.07	C_21_H_30_O_11_	381.1240, 281.1407, 223.0991, 205.0880, 175.0270	457.1709	457.1715	−1.4	+2O, +GlcUA	−	+	−
M21	15.18	C_21_H_30_O_11_	381.1298, 205.0890	457.1701	457.1715	−3.1	+2O, +GlcUA isomer 2	−	+	−
M22	16.71	C_21_H_30_O_11_	439.1619, 381.1219, 205.0873	457.1712	457.1715	−0.7	+2O, +GlcUA isomer 3	−	+	−
M23	17.87	C_21_H_30_O_11_	439.1640, 205.0892, 175.0268	457.1704	457.1715	−2.5	+2O, +GlcUA	−	+	−
M24	26.65	C_15_H_20_O_3_	189.0938, 173.0624, 147.0749, 131.0511	247.1342	247.1340	0.9	+O, −H_2_O	+	+	−
M25	15.61	C_15_H_20_O_4_	245.1151	263.1281	263.1289	−3.0	+O, −2H isomer 1	−	+	−
M26	18.58	C_15_H_20_O_4_	245.1219, 231.1029	263.1291	263.1289	0.8	+O, −2H isomer 2	+	+	−
M27	23.69	C_15_H_20_O_4_	245.1211, 205.0915, 161.0617	263.1291	263.1289	0.8	+O, −2H isomer 3	+	+	−
M28	19.79	C_15_H_20_O_4_	205.0871, 189.0562, 175.0409, 147.0456	263.1289	263.1289	0.1	+O, −2H isomer 4	+	+	−
M29	24.33	C_15_H_20_O_4_	205.0877, 189.0540, 175.0436	263.1287	263.1289	−0.7	+O, −2H isomer 5	+	+	−
M30	22.82	C_15_H_20_O_4_	205.0873, 175.0444	263.1291	263.1289	0.8	+O, −2H isomer 6	−	+	−
M31	11.21	C_15_H_20_O_5_	221.0817	279.1236	279.1238	−0.7	+2O, −2H isomer 7	−	+	−
M32	12.14	C_15_H_20_O_5_	221.0867, 179.0698, 163.0409	279.1232	279.1238	−2.1	+2O, −2H isomer 8	−	+	−
M33	14.56	C_15_H_20_O_5_	205.0871, 179.0725, 163.0417, 165.0495	279.1238	279.1238	0.0	+2O, −2H isomer 9	−	+	−
M34	24.56	C_15_H_20_O_5_	261.1149	279.1236	279.1238	−0.7	+2O, −2H isomer 10	−	+	−
M35	15.81	C_15_H_20_O_5_	221.0897, 205.0871, 163.0397, 165.0570	279.1241	279.1238	1.1	+2O, −2H isomer 11	−	+	+
M36	22.83	C_15_H_20_O_5_	221.0986	279.1230	279.1238	−2.9	+2O, −2H isomer 12	+	−	−
M37	28.87	C_15_H_20_O_5_	221.1196, 163.0133	279.1231	279.1238	−2.5	+2O, −2H isomer 13	−	+	−
M38	12.12	C_21_H_28_O_11_	279.1267, 261.1109, 221.0843, 175.0760, 175.0242	455.1553	455.1559	−1.3	+2O, −2H, +GlcUA isomer 1	−	+	−
M39	13.87	C_21_H_28_O_11_	397.1145, 279.1180, 221.0838	455.1544	455.1559	−3.3	+2O, −2H, +GlcUA isomer 2	+	+	−
M40	11.37	C_21_H_28_O_11_	455.1558, 175.0251	455.1553	455.1559	−1.3	+2O, −2H, +GlcUA isomer 3	+	−	−
M41	22.62	C_15_H_18_O_4_	203.0736, 187.0420, 173.0268	261.1140	261.1132	2.9	+2O, −2H, −H_2_O isomer 4	+	+	−
M42	10.78	C_15_H_20_O_6_	223.1352	295.1181	295.1187	−2.1	+3O, −2H isomer 1	+	−	+
M43	18.57	C_15_H_20_O_6_	277.1086, 251.1299, 233.1187, 205.1245	295.1192	295.1187	1.7	+3O, −2H isomer 2	+	+	+
M44	23.86	C_15_H_20_O_6_	251.1338, 205.1256	295.1185	295.1187	−0.7	+3O, −2H isomer 3	−	+	−
M45	11.42	C_16_H_24_O_6_	151.9993, 124.0063, 106.9804	311.1494	311.1500	−2.0	+O, +2H, +CO_2_ isomer 1	+	−	−
M46	12.40	C_16_H_24_O_6_	229.1253, 189.0977	311.1496	311.1500	−1.3	+O, +2H, + CO_2_ isomer 2	−	+	−
M47	19.87	C_16_H_24_O_6_	267.1557, 247.1354, 229.1216, 205.1249, 189.0960, 163.0742, 147.0488	311.1500	311.1500	0.0	+O, +2H, + CO_2_ isomer 3	+	+	+
M48	17.44	C_16_H_24_O_6_	247.1393, 205.1184, 189.0912, 149.0603	311.1498	311.1500	−0.7	+O, +2H, + CO_2_ isomer 4	−	+	−
M49	20.55	C_21_H_30_O_9_	193.0368, 175.0230, 131.0366, 117.0200, 115.0023, 113.0253, 101.0242	425.1808	425.1817	−2.1	+GlcUA	+	+	−
M50	23.16	C_21_H_30_O_9_	367.1381, 191.1079, 175.0247	425.1804	425.1817	−3.1	+GlcUA	+	+	−
M51	17.99	C_21_H_30_O_9_	367.1411, 191.1080, 175.0223, 175.0758	425.1882	425.1817	−3.5	+GlcUA	+	+	−
M52	25.38	C_21_H_30_O_9_	175.0255	425.1810	425.1817	−1.7	+GlcUA	+	−	−
M53	28.31	C_21_H_30_O_9_	231.1406, 215.1077	425.1814	425.1817	−0.7	+GlcUA isomer 1	−	+	−
M54	10.12	C_21_H_32_O_10_	351.0681, 193.0358, 175.0265, 157.0157	443.1925	443.1923	0.5	+OH, +2H, +GlcUA isomer 1	+	−	−
M55	12.18	C_21_H_32_O_10_	385.1545, 367.1391, 341.1277, 193.0365, 191.1083, 165.0930	443.1921	443.1923	−0.4	+OH, +2H, +GlcUA isomer 2	+	+	−
M56	16.03	C_21_H_32_O_10_	191.1089, 175.0257	443.1920	443.1923	−0.6	+OH, +2H, +GlcUA isomer 3	+	+	−
M57	10.90	C_12_H_16_O_4_	191.1133, 161.1032, 159.0869	223.0974	223.0974	−0.8	−C_3_H_6_O, +2O	+	−	−
M58	24.29	C_12_H_14_O_3_	190.0677, 175.0420	205.0871	205.0870	0.4	−C_3_H_6_O, +2O, −H_2_O isomer 1	−	+	−
M59	22.67	C_12_H_14_O_3_	190.0665, 175.0436	205.0862	205.0870	−4.0	−C_3_H_6_O, +2O, −H_2_O isomer 2	−	+	−
M60	21.74	C_12_H_14_O_3_	190.0634, 177.0923, 175.0410, 147.0455	205.0873	205.0870	1.4	−C_3_H_6_O, +2O, −H_2_O isomer 3	−	+	−
M61	19.79	C_12_H_14_O_3_	190.0648, 175.0404, 147.0457	205.0867	205.0870	−1.6	−C_3_H_6_O, +2O, −H_2_O isomer 4	+	+	−
M62	15.56	C_18_H_22_O_9_	205.0886, 190.0663, 175.0436	381.1183	381.1191	−2.1	−C_3_H_6_O, +2O, −H_2_O, +GlcUA	−	+	−
M63	23.83	C_18_H_24_O_8_	193.0365, 191.1084	367.1380	367.1398	−5.0	−C_3_H_6_O, +GlcUA isomer 1	−	+	−
M64	24.84	C_18_H_24_O_8_	193.0373, 191.1059, 175.0774, 175.0220	367.1393	367.1398	−1.5	−C_3_H_6_O, +GlcUA isomer 2	+	+	−
M65	25.31	C_18_H_24_O_8_	191.1084, 175.0823, 161.0620, 149.0641	367.1392	367.1398	−1.7	−C_3_H_6_O, +GlcUA isomer 3	−	+	−
M66	26.68	C_12_H_16_O_5_S	191.1075, 173.0987, 157.0648	271.0636	271.0646	−1.7	−C_3_H_6_O, +SO_3_ isomer 4	+	+	−
M67	19.76	C_12_H_16_O_5_S	189.0951	271.0638	271.0646	−2.8	−C_3_H_6_O, +SO_3_ isomer 5	+	−	−
M68	11.99	C_12_H_16_O_5_S	96.9618, 80.9671	271.0650	271.0646	1.6	−C_3_H_6_O, +SO_3_ isomer 6	−	−	+
M69	20.61	C_12_H_16_O_5_S	80.9641	271.0651	271.0646	2.0	−C_3_H_6_O, +SO_3_ isomer 7	−	−	+
M70	23.99	C_12_H_16_O_5_S	191.1113, 96.9606, 80.9665	271.0644	271.0646	−0.6	−C_3_H_6_O, +SO_3_ isomer 8	−	−	+
M71	19.03	C_12_H_18_O_5_S	165.0979, 80.9648	273.0797	273.0802	−1.9	−C_3_H_6_O, +2H, +SO_3_ isomer 1	−	−	+
M72	10.66	C_12_H_18_O_5_S	80.9642	273.0806	273.0802	1.4	−C_3_H_6_O, +2H, +SO_3_ isomer 2	−	−	+
M73	23.52	C_12_H_18_O_5_S	191.1087, 80.9654	273.0804	273.0802	0.7	−C_3_H_6_O, +2H, +SO_3_ isomer 3	−	−	+
M74	16.37	C_12_H_18_O_5_S	191.1042, 80.9644	273.0813	273.0802	4.0	−C_3_H_6_O, +2H, +SO_3_ isomer 4	−	+	+
M75	15.69	C_12_H_18_O_5_S	80.9650	273.0803	273.0802	0.3	−C_3_H_6_O, +2H, +SO_3_ isomer 5	+	−	−
M76	9.96	C_12_H_18_O_5_S	191.1131, 80.9652	273.0799	273.0802	−1.2	−C_3_H_6_O, +2H, +SO_3_ isomer 6	−	−	+

^1^ t_R_, retention time; Meas., measured; Pred., predicted; U, urine; F, feces; P, plasma; +, detected; −, undetected. ^2^ original compound of nardosinone.

## Data Availability

Not applicable.

## Supplementary Materials

The following supporting information can be downloaded at: https://www.mdpi.com/article/10.3390/molecules27217267/s1, Figure S1: Extracted ion chromatograms (EICs) of 39 metabolites of nardosinone in mice urine. A, B, C, and D represent the EICs of 39 metabolites of nardosinone group, the magnified EICs of 39 metabolites of nardosinone group, the EICs of 39 metabolites of blank group, the magnified EICs of 39 metabolites of blank group, respectively. Figure S2: Extracted ion chromatograms (EICs) of 51 metabolites of nardosinone in mice plasma. A, B, C, and D represent the EICs of 51 metabolites of nardosinone group, the magnified EICs of 51 metabolites of nardosinone group, the EICs of 51 metabolites of blank group, the magnified EICs of 51 metabolites of blank group, respectively. Figure S3: Extracted ion chromatograms (EICs) of 12 metabolites of nardosinone in mice feces. A and B represent the EICs of 12 metabolites of nardosinone group and blank group, respectively. Figure S4: The possible structures and proposed fragmentation pathways of M66-M70. The red rim represents the proposed fragmentation pathways of M67-M70 and the blue rim represents the proposed fragmentation pathway of M66. Figure S5: The possible structures and proposed fragmentation pathways of M71-M76. The olive rim represents the possible structures. The dull purple rim represents the proposed fragmentation pathways. Figure S6: Extracted ion chromatograms (EICs) of *m*/*z* 273.08.
