# Supplementary material for: Analysis of In Vivo Existence Forms of Nardosinone in Mice by UHPLC-Q-TOF-MS Technique
Source: Molecules. 2022 Oct 26;27(21):7267. doi: 10.3390/molecules27217267 (PMC9653913; doi:10.3390/molecules27217267)
Supplement: Supplementary file 1 [file molecules-27-07267-s001.zip › Figure S4 and Figure S5.pdf]

Supplementary materials

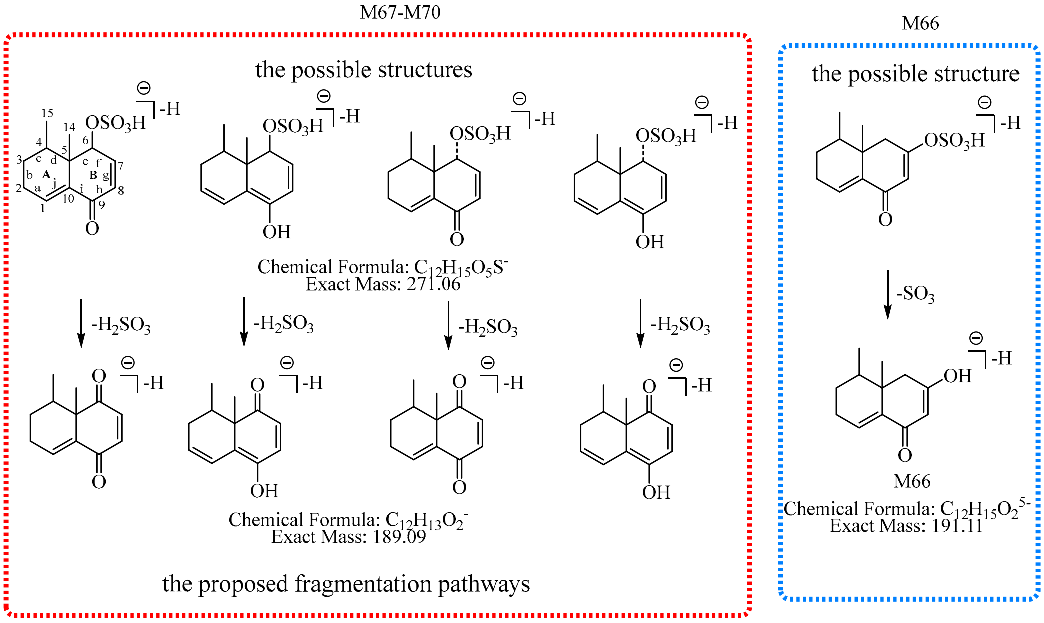

Figure S4. The possible structures and proposed fragmentation pathways of M66-M70. The red rim represents the proposed fragmentation pathways of M67-M70 and the blue rim represents the proposed fragmentation pathway of M66.

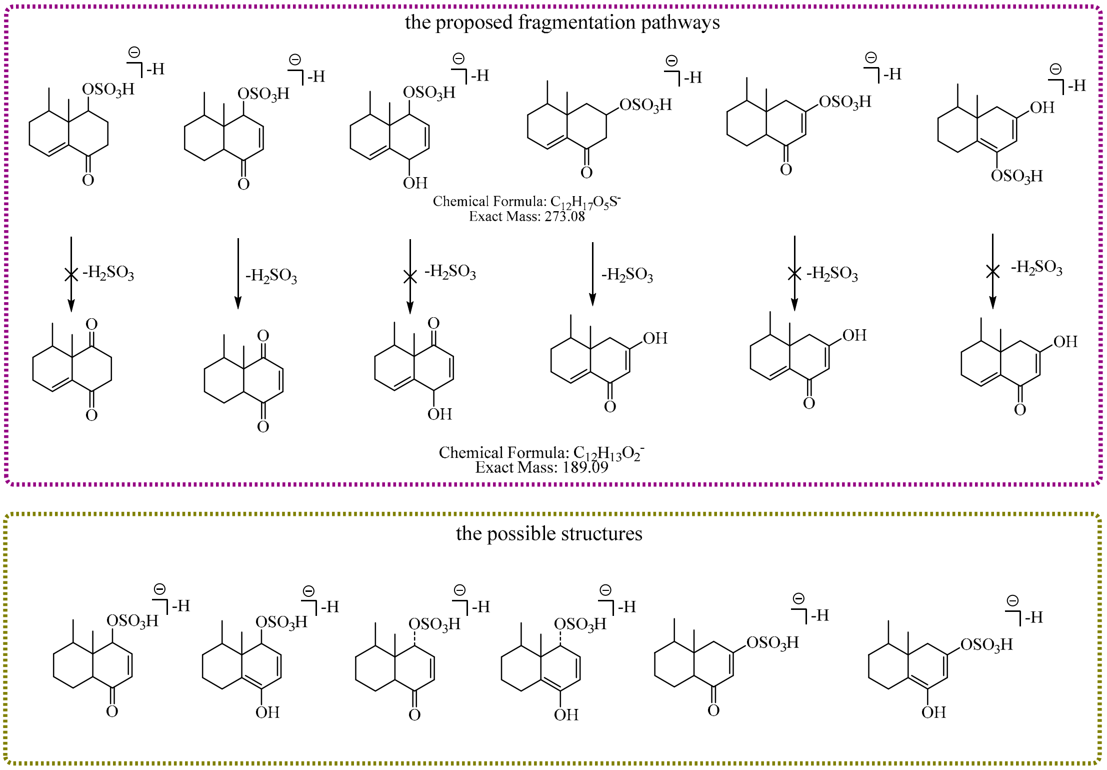

Figure S5. The possible structures and proposed fragmentation pathways of M71-M76. The olive rim represents the possible structures. The dull purple rim represents the proposed fragmentation pathway
