# Supplementary figures and images for: Analysis of In Vivo Existence Forms of Nardosinone in Mice by UHPLC-Q-TOF-MS Technique
Source: Molecules. 2022 Oct 26;27(21):7267. doi: 10.3390/molecules27217267 (PMC9653913; doi:10.3390/molecules27217267)

A

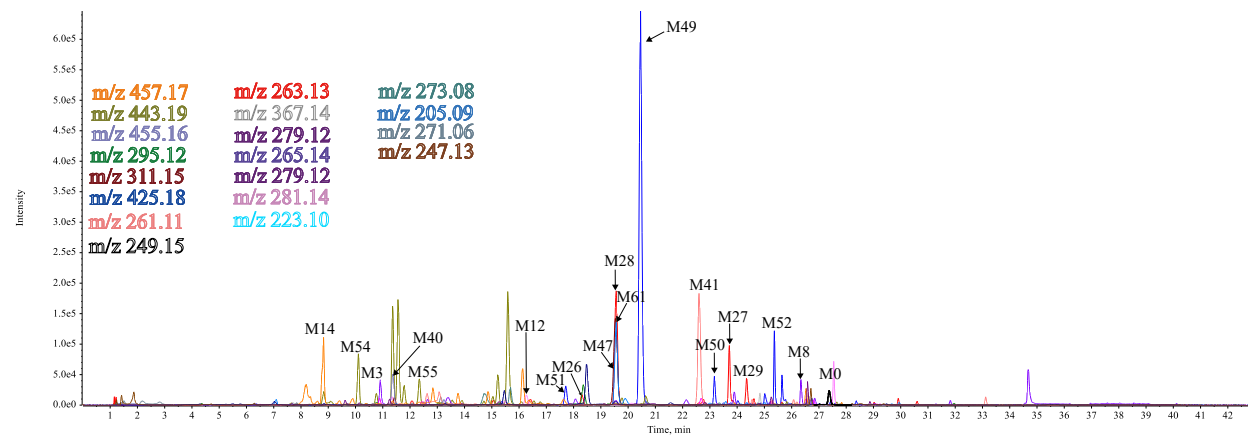

B

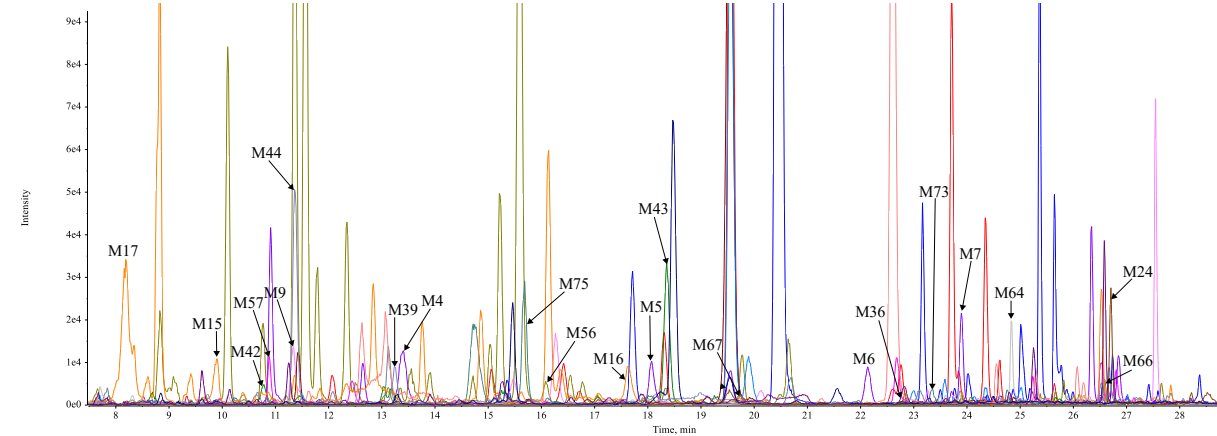

C

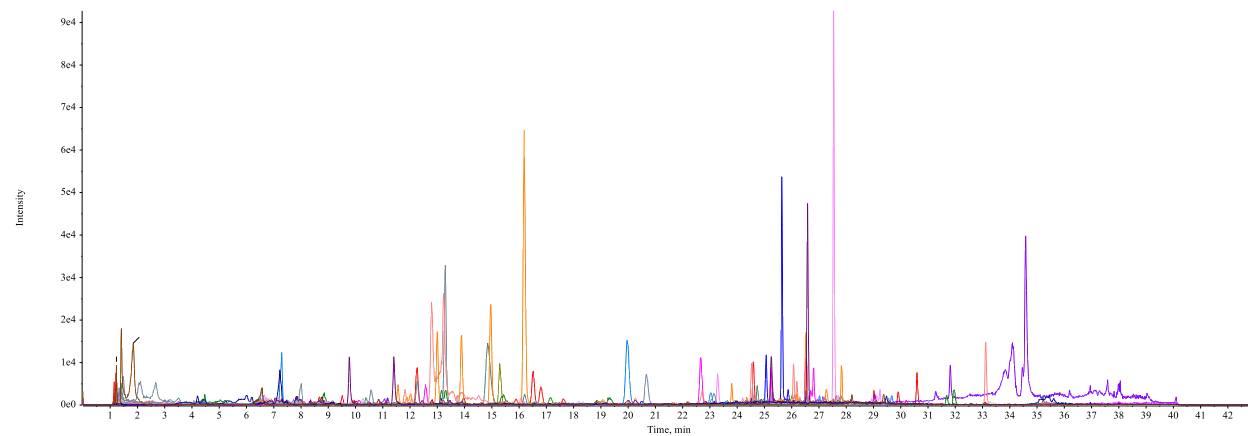

D

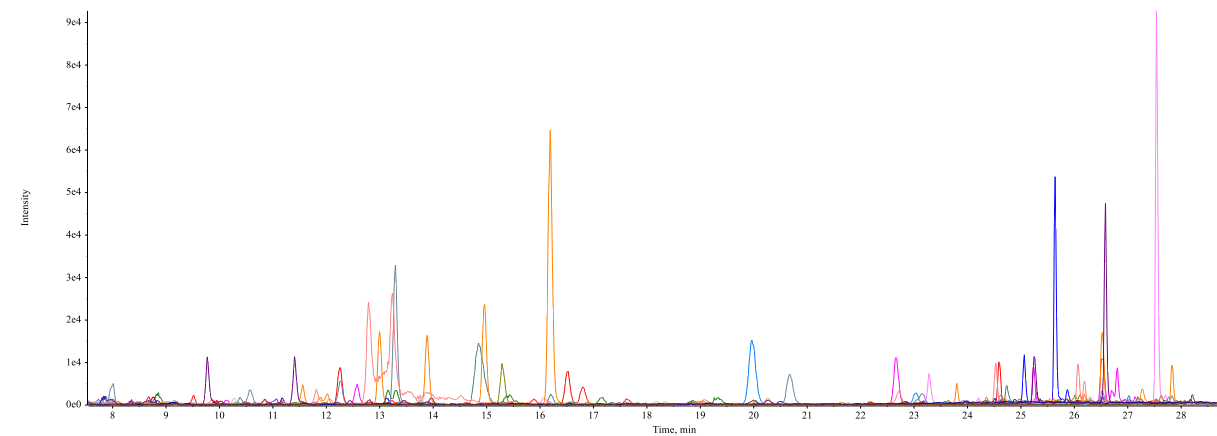

Supplement: Supplementary file 1 [file molecules-27-07267-s001.zip › Figure S1.pdf]

A

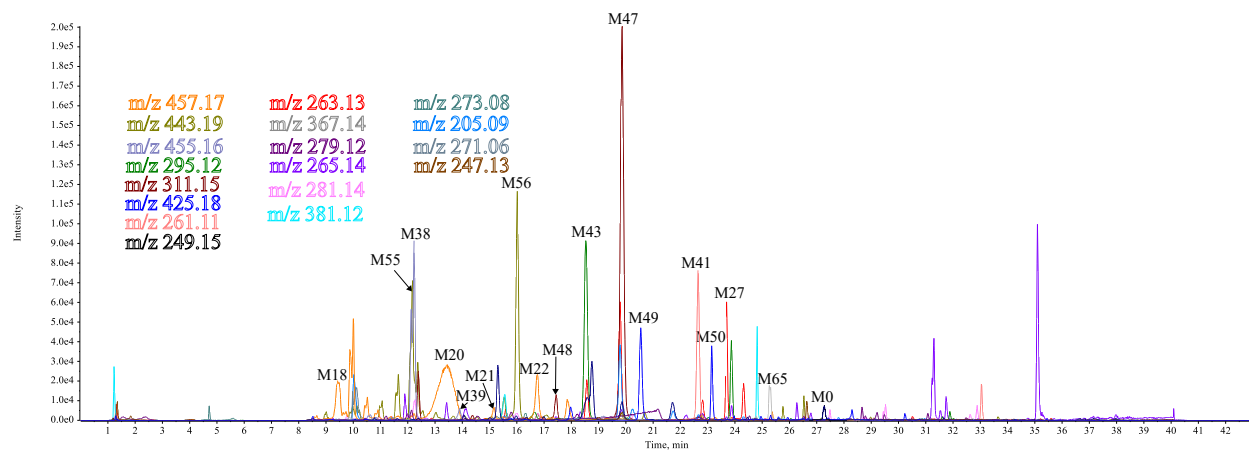

B

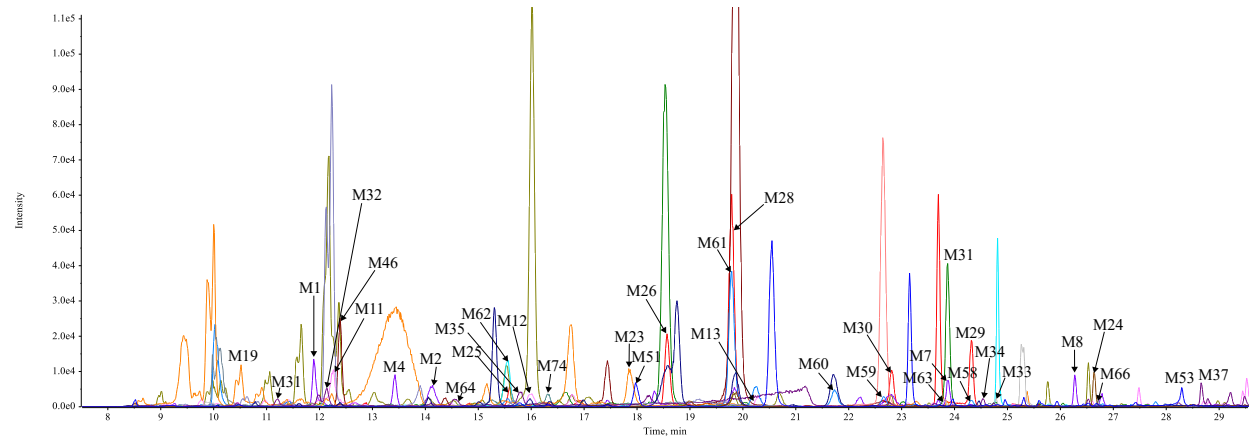

C

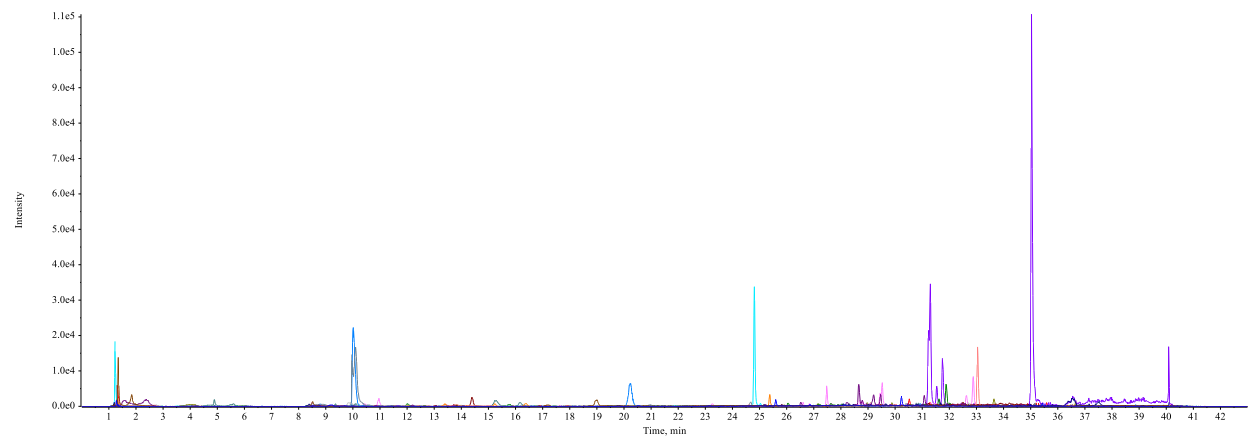

D

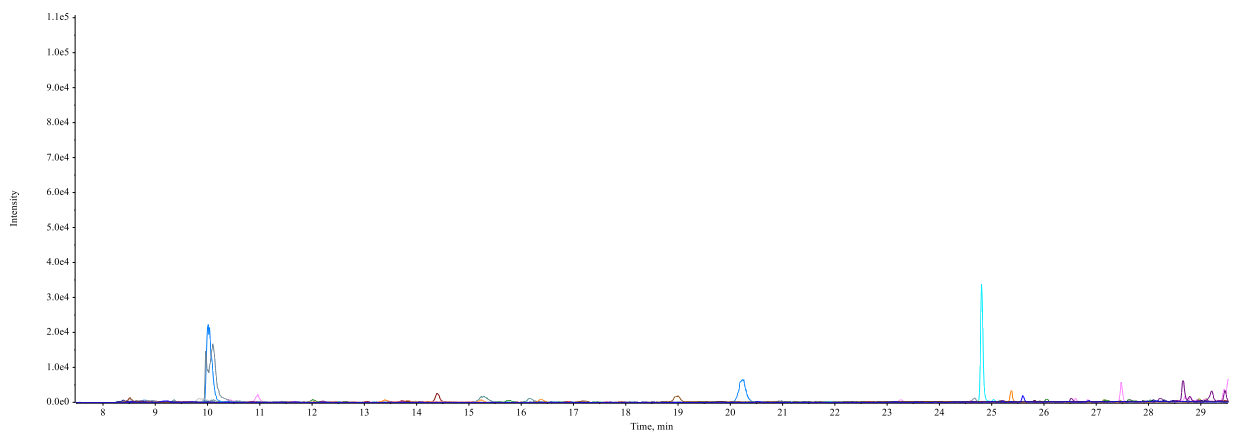

Supplement: Supplementary file 1 [file molecules-27-07267-s001.zip › Figure S2.pdf]

A

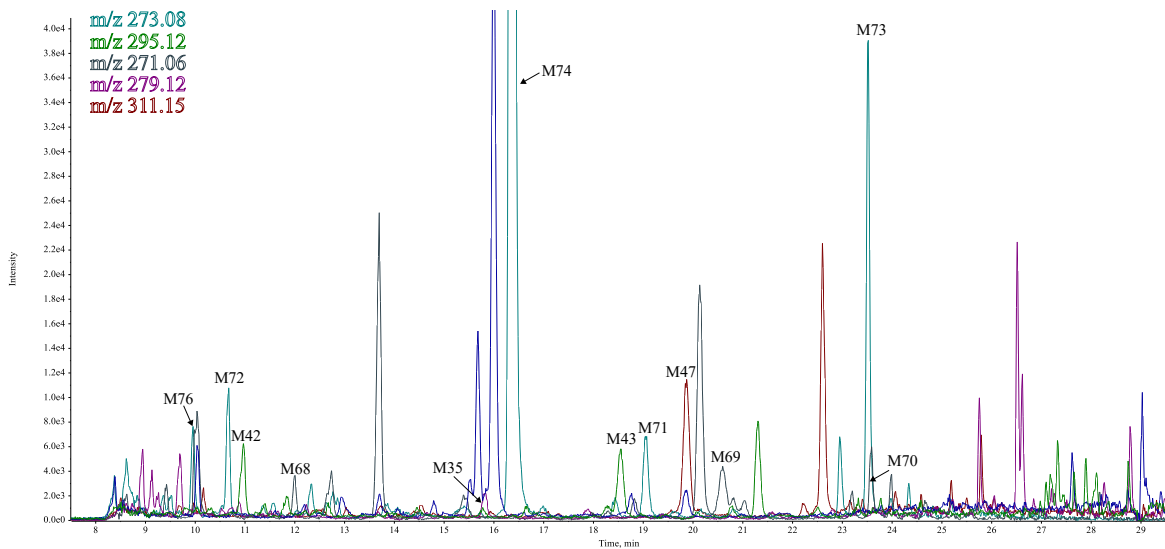

B

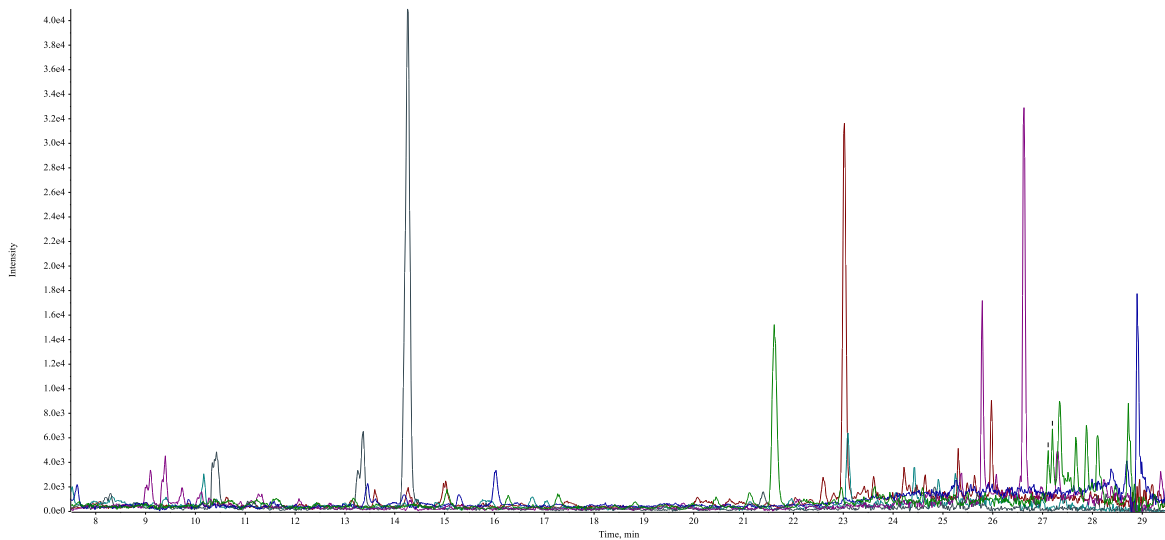

Supplement: Supplementary file 1 [file molecules-27-07267-s001.zip › Figure S3.pdf]

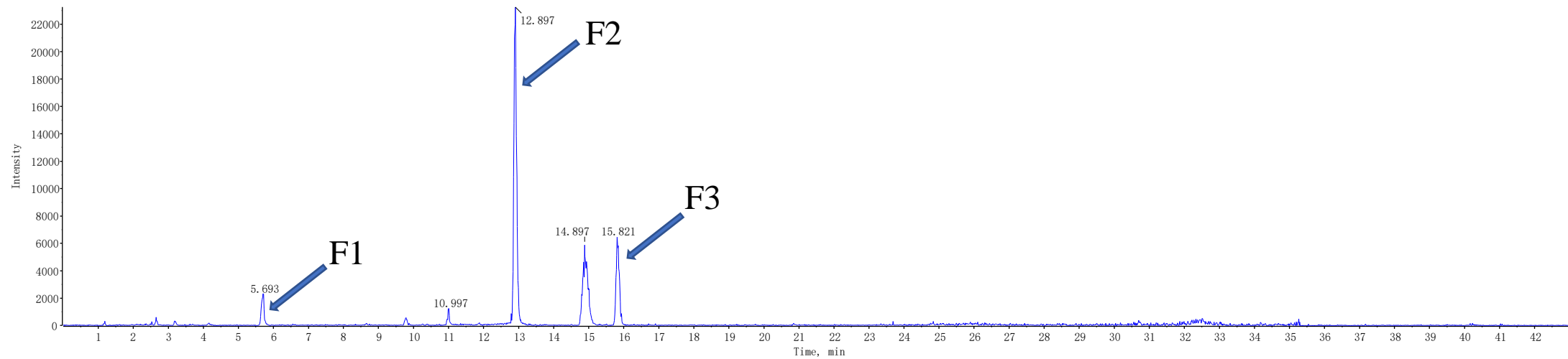

Figure S6. Extracted ion chromatogram (EIC) of  $m/z$  273.08.

Supplement: Supplementary file 1 [file molecules-27-07267-s001.zip › Figure S6.pdf]
